# Concurrent intrathecal and intravenous nivolumab in leptomeningeal disease: phase 1 trial interim results

**DOI:** 10.1038/s41591-022-02170-x

**Published:** 2023-03-30

**Authors:** Isabella C. Glitza Oliva, Sherise D. Ferguson, Roland Bassett, Alexandra P. Foster, Ida John, Tarin D. Hennegan, Michelle Rohlfs, Jessie Richard, Masood Iqbal, Tina Dett, Carol Lacey, Natalie Jackson, Theresa Rodgers, Suzanne Phillips, Sheila Duncan, Lauren Haydu, Ruitao Lin, Rodabe N. Amaria, Michael K. Wong, Adi Diab, Cassian Yee, Sapna P. Patel, Jennifer L. McQuade, Grant M. Fischer, Ian E. McCutcheon, Barbara J. O’Brien, Sudhakar Tummala, Matthew Debnam, Nandita Guha-Thakurta, Jennifer A. Wargo, Fernando C. L. Carapeto, Courtney W. Hudgens, Jason T. Huse, Michael T. Tetzlaff, Elizabeth M. Burton, Hussein A. Tawbi, Michael A. Davies

**Affiliations:** 1 Department of Melanoma Medical Oncology, The University of Texas MD Anderson Cancer Center, Houston, TX USA; 2 Department of Neurosurgery, The University of Texas MD Anderson Cancer Center, Houston, TX USA; 3 Department of Biostatistics, The University of Texas MD Anderson Cancer Center, Houston, TX USA; 4 Department of Surgical Oncology, The University of Texas MD Anderson Cancer Center, Houston, TX USA; 5 Department of Neuro-Oncology, The University of Texas MD Anderson Cancer Center, Houston, TX USA; 6 Department of Neuroradiology, The University of Texas MD Anderson Cancer Center, Houston, TX USA; 7 Department of Translational Molecular Pathology, The University of Texas MD Anderson Cancer Center, Houston, TX USA; 8 Department of Pathology, The University of Texas MD Anderson Cancer Center, Houston, TX USA; 9 Department of Pathology, The University of California San Francisco, San Francisco, CA USA; 10 Department of Genomic Medicine, The University of Texas MD Anderson Cancer Center, Houston, TX USA

**Keywords:** Cancer immunotherapy, Phase I trials, CNS cancer, Melanoma

## Abstract

There is a critical need for effective treatments for leptomeningeal disease (LMD). Here, we report the interim analysis results of an ongoing single-arm, first-in-human phase 1/1b study of concurrent intrathecal (IT) and intravenous (IV) nivolumab in patients with melanoma and LMD. The primary endpoints are determination of safety and the recommended IT nivolumab dose. The secondary endpoint is overall survival (OS). Patients are treated with IT nivolumab alone in cycle 1 and IV nivolumab is included in subsequent cycles. We treated 25 patients with metastatic melanoma using 5, 10, 20 and 50 mg of IT nivolumab. There were no dose-limiting toxicities at any dose level. The recommended IT dose of nivolumab is 50 mg (with IV nivolumab 240 mg) every 2 weeks. Median OS was 4.9 months, with 44% and 26% OS rates at 26 and 52 weeks, respectively. These initial results suggest that concurrent IT and IV nivolumab is safe and feasible with potential efficacy in patients with melanoma LMD, including in patients who had previously received anti-PD1 therapy. Accrual to the study continues, including in patients with lung cancer. ClinicalTrials.gov registration: NCT03025256.

## Abstract (web summary)

In an interim analysis of a phase 1 trial of concurrent intrathecal and intravenous anti-PD1 delivery in patients with leptomeningeal disease and melanoma, treatment was feasible and well-tolerated with no dose-limiting toxicities.

## Main

Melanoma has the highest propensity among all common solid tumors for the development of central nervous system (CNS) metastases^1^. Although recent breakthroughs with systemic therapies have shown encouraging results for melanoma patients with extracranial disease only, as well as parenchymal melanoma brain metastases (MBM), there has been little progress for patients with LMD^2–6^. LMD from melanoma is associated with a high neurological symptom burden, and the historical median OS is approximately 6 weeks^7^. With recent advances in immune and targeted therapies prolonging the survival of patients with metastatic melanoma, as well as CNS surveillance with magnetic resonance imaging (MRI), the overall incidence of LMD, often considered a late disease complication, is rising^8^.

There is a clear unmet need for new, more effective treatments for melanoma patients with LMD. The current therapeutic options for melanoma LMD patients consist of radiation, systemically administered therapies or a limited number of IT therapies^7,9^. Most available data assessing outcomes have been from retrospective studies because there have been very few prospective trials conducted in this patient population. Whole-brain radiation therapy can provide palliative relief but does not impact OS^4^. Systemic administration of chemotherapy has failed to demonstrate significant efficacy in the treatment of LMD from melanoma^10^. For patients with a *BRAF*^*V600*^ mutation, targeted therapy with combinations of BRAF and MEK inhibitors can result in clinical responses, as described in case reports and small retrospective series^7^. However, no prospective evaluation in LMD patients has been performed. Immune checkpoint inhibitors have demonstrated durable benefit in patients with metastatic melanoma, including in patients with parenchymal brain metastases, in multiple prospective clinical trials^5,11–13^. Treatment of patients with asymptomatic MBMs with single-agent ipilimumab, pembrolizumab or nivolumab achieved objective intracranial response rates (OICRR) of 18%, 20% and 20%, respectively; but importantly, all MBM trials excluded patients with previous immune checkpoint inhibitor treatment^2,12,13^. Higher activity has been observed with ipilimumab and nivolumab in patients with asymptomatic MBMs, with OICRR of 55% in the CheckMate-204 trial (*n* = 101 patients) and 51% in the ABC trial (*n* = 35)^2,5^. Notably, in all of these studies, the overwhelming majority of intracranial responses were durable, and responding patients had 3-year survival over 90%^5^. Much lower OICRR were observed in patients with symptomatic MBMs with single-agent ipilimumab (5%) and with ipilimumab + nivolumab (22%). Importantly, all but the ABC trial excluded patients with LMD, and none of the four patients with LMD included in the exploratory cohort and treated with single-agent IV nivolumab responded.

Owing to the poor penetration of many agents into the cerebrospinal fluid (CSF), IT administration of anti-cancer agents has been explored in patients with LMD in multiple cancer types. IT administration of trastuzumab in breast cancer and of rituximab in lymphoma patients demonstrated that such approaches achieve significant drug exposure in the CSF and clinical benefit in patients with LMD, thus frequently being used in this setting, although randomized trials are still needed to definitively assess the efficacy of this approach^14^. In melanoma, IT interleukin-2 (IL-2) has been explored as an immunotherapeutic approach for LMD since the early 1990s^15^. Similar to systemic IL-2, only a subset of patients will experience a durable benefit from this approach, but we recently reported an encouraging median OS of 7.8 months and a 5-year OS of 13% for IT IL-2 treatment in a cohort of highly selected melanoma LMD patients, and provided initial evidence that IT immunotherapy can potentially result in long-term survival in a subset of patients with LMD^16^. However, patients treated with IT IL-2 require prolonged hospitalization (~4 weeks) as well as monitoring and assessment by a highly specialized team to manage the serious toxicities of IT IL-2 related to meningeal inflammation and increased intracranial pressure (headache, nausea, vomiting, change in mental status, neck stiffness, decrease in overall performance status). Thus, this approach is not feasible outside highly specialized treatment settings and is not a viable option for most patients. Despite its limited use owing to toxicities, the results with IT IL-2 demonstrate that long-term disease control and survival in melanoma LMD patients is possible with IT immunotherapy and warrants further investigation with more modern immunotherapy agents.

Based on the improved efficacy and safety of systemically administered anti-PD1 compared with IL-2, we hypothesized that the treatment of patients with melanoma LMD with IT anti-PD1 would be safe and feasible. We first evaluated the toxicity of IT administration of anti-PD1 antibody in an immunocompetent mouse model. We then designed a first-in-human dose-finding study using concurrent administration of IT and IV anti-PD1. Here, we report a nonprespecified interim analysis detailing the dose escalation portion, recommended IT dose, safety profile and preliminary signal for efficacy of concurrent IT and IV nivolumab in melanoma patients with LMD.

## Results

### Preclinical safety evaluation of IT anti-PD1

Because no clinical studies had previously evaluated IT administration of anti-PD1, we first tested this approach in immunocompetent female mice to evaluate safety. For a detailed summary of the animal dosing, please refer to the Methods.

**Extended Data Fig. 1:**
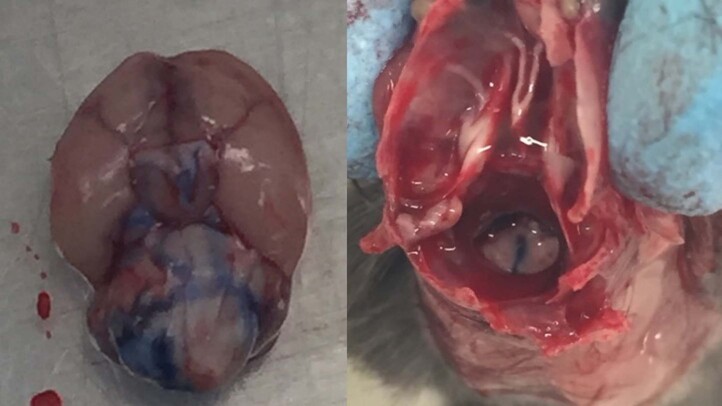
Confirmation of cisternal injection technique. Methylene blue dye delivered via subcisternal injection readily collected in the subarachnoid space (A) and spinal cord canal (B), confirming successful penetration of the leptomeninges.

**Extended Data Fig. 2:**
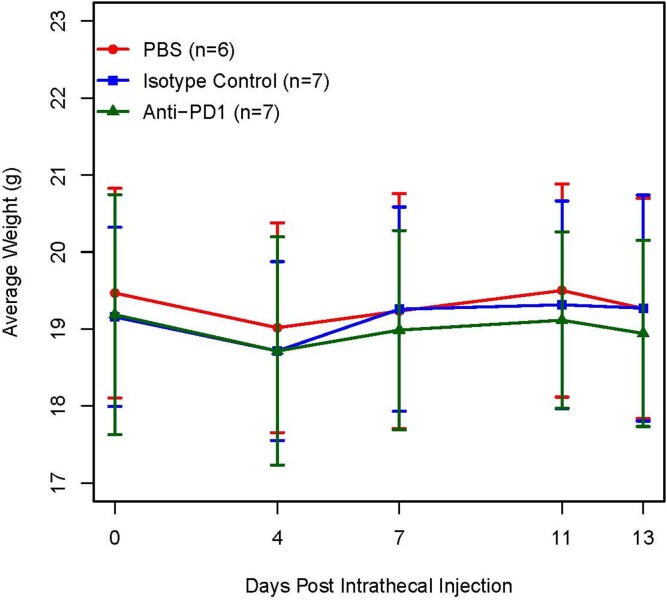
Safety evaluation of IT anti-PD1 in C57BL/6 mice. Twenty mice were divided into three volume matched (6.5μl) treatment groups, 1). IT PBS (N=6); 2). IT isotype control (N = 7); 3). IT mouse anti-PD1 (13μg) (N = 7). Line graph plotting the mean weight of animals in the three IT treatment groups. Bars represent standard errors of the mean. No mice displayed morbidity/significant weight change during 14-day observation period. Note that additional animals were observed at 48 hours (PBS = 7; IT isotype control = 8; IT anti-PD1 = 8) and 7 day (PBS = 5; IT isotype control= 7; IT anti-PD-1 = 7) time points (prior to euthanasia for histological analysis) and no significant weight changes were observed at these time points.

**Extended Data Fig. 3:**
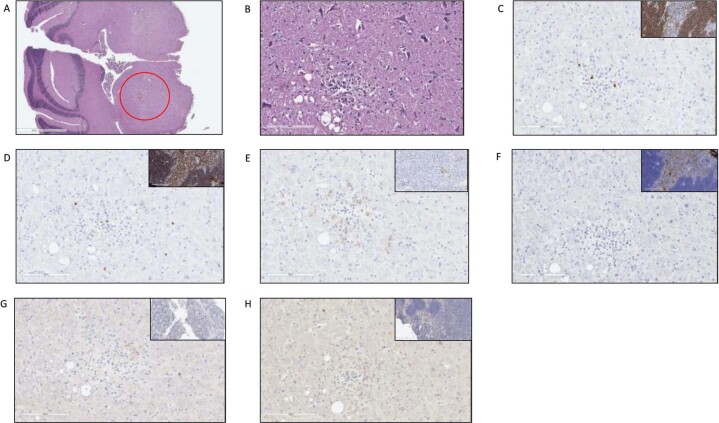
Assessment of inflammatory response after IT administration of either PBS, Isotype or anti-PD1. Sample mouse brain parenchyma pathology 7 days following intrathecal injection of anti-PD1 antibody (6.5 µl). A and B: H & E, 2x (A) and 20x (B). C-H staining for CD8 (C), CD3 (D), PD1 (E), PD-L1 (F), CD15 (G) and CD163 (H). Upper right insert represents positive control. Of note, no evidence of treatment related inflammation was noted at the 48 hours, 7 days and 14 days timepoint (representative data shown).

We evaluated an IT dose of 13 μg of murine anti-PD1 antibody, which was equivalent to a dose of 50 mg in patients based on differences in CSF volumes in mice versus humans. The dose was administered in a volume of 6.5 μl via the cisterna magna and was compared with the effects of IT administration of equal volumes of vehicle or rat immunuglobulin-G2a (IgG2a) control antibodies (vehicle, *n* = 22; isotype antibody, *n* = 23; anti-PD1, *n* = 23). Analysis showed methylene blue dye in the subarachnoid space and spinal canal post injection, confirming that the dye successfully circulated through the CSF space (Extended Data Fig. 1). Six mice died during the injection procedure (perioperative mortality rate 9.7%): 4 of 22 in the phosphate-buffered saline (PBS) group, 1 of 23 in the isotype group and 1 of 23 in the anti-PD1 group. The remaining animals were euthanized at 2, 7 and 14 d after IT injection. During this period no mice displayed morbidity, neurological symptoms or extreme (>20%) weight loss, and mice had daily assessments and were weighed twice per week (Extended Data Fig. 2). Brain and spinal cord tissue collected at all time points were evaluated by immunohistochemistry (IHC) for expression of CD3, CD8, CD163, CD56, CD15, PD1 and PD-L1. Histologic review showed no evidence of neurological damage other than mechanical effects observed at the injection site with all treatments (representative IHC images are in Extended Data Fig. 3). There were no differences in immune markers between the treatment groups. For the 48 h time point, CNS tissue from 20 mice was assessed for inflammation (PBS, *n* = 6; isotype, *n* = 7; anti-PD1, *n* = 7), from 19 mice at the 7 d time point (PBS, *n* = 5; isotype, *n* = 7; anti-PD1, *n* = 7) and from 19 mice at the 14 d time point (PBS, *n* = 5; isotype, *n* = 7; anti-PD1, *n* = 7). With this preclinical demonstration of safety, we proceeded with our first-in-human study.

### Clinical trial design

**Fig. 1:**
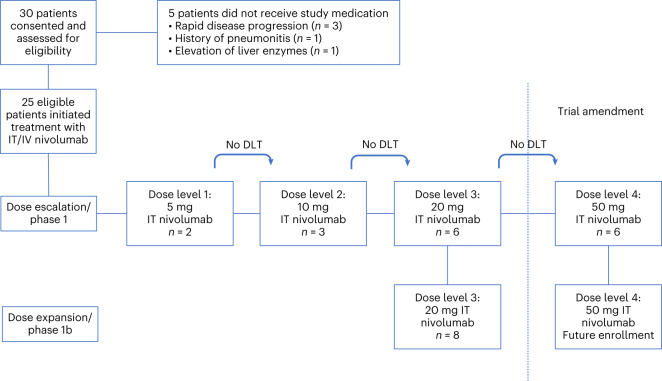
Overview of enrollment into the different dose levels and phases of the study. The study consisted of two parts: a phase 1 dose escalation portion and a phase 1b dose expansion cohost at the recommended dose. This graph details the patients enrolled on each dose level (5, 10, 20 and 50 mg after protocol amendment) and phase (dose escalation and dose expansions) of the clinical trial. A total of 25 patients were treated.

We designed a phase 1/1b study of concurrent IT and IV nivolumab in melanoma patients with LMD. The study consists of two parts: an IT dose escalation portion and a dose expansion cohort at the maximum tolerated dose (MTD) (Fig. 1).

The initial enrollment plan, based on three IT nivolumab dose levels (5, 10 and 20 mg), anticipated a maximum of 18 patients in the dose escalation part, and 12 additional patients in the dose expansion part (*n* = 30). Due to the lack of observed toxicities, the trial protocol was then amended and 6 patients were treated with 50 mg (Fig. 1), and the enrollment plan was modified to enroll no more than 17 patients in the dose escalation part and 33 patients in the dose expansion part (*n* = 50).

Eligible patients are 18 years or older and have histologically confirmed melanoma, including all subtypes (cutaneous, acral lentiginous, mucosal, uveal, primary CNS or unknown primary), without any history of allergy to the study drug components or severe hypersensitivity to any monoclonal antibody. LMD is confirmed by CSF cytopathology and/or consistent findings on MRI brain/spine as confirmed by a neuroradiologist. Patients are required to have an Eastern Cooperative Oncology Group (ECOG) performance status of 2 or lower, have acceptable organ and marrow function, and are on a stable or decreasing dose of dexamethasone (≤4 mg per 24 h). Previous therapy directed at LMD and/or metastatic melanoma is allowed, including IT therapy (washout 7 d), radiation (washout 7 d) and systemic biologic therapy (CTLA4 and/or PD, IL-2, interferon, washout 14 d). Any prior dose of chemotherapy or investigational therapy has to have been given at least 14 d prior to the first dose of IT nivolumab. Patients must be capable of giving written informed consent, which includes compliance with the study requirements.

Patients are not eligible if they have a history of HIV or AIDS, acute or chronic hepatitis B or C infection, previous anti-PD1 therapy-induced pneumonitis, or have ongoing >Grade 2 adverse events of such therapy; or ongoing autoimmune disease that required systemic treatment in the past 2 years. In addition, pregnant or lactating females are not eligible, as well as patients with a major medical, neurologic or psychiatric condition who are judged as unable to fully comply with study therapy or assessments. Evidence of active infection ≤7 d before initiation of study drug therapy or use of nononcology vaccines containing live virus for prevention of infectious diseases within 12 weeks before study drug therapy renders participants ineligible. Finally, prisoners or patients who are involuntarily incarcerated and patients who are compulsorily detained for treatment of either a psychiatric or physical (e.g. infectious disease) illness are not eligible.

From 7 May 2018 to 22 January 2021, a total of 30 metastatic melanoma patients with LMD were consented and enrolled. The data cutoff for this analysis was 6 June 2021. Five patients did not receive any study medication: three experienced rapid disease progression during screening when targeted therapy was stopped for trial initiation, one was found to be ineligible for history of pneumonitis and another was ineligible for elevation in liver enzymes. The remaining patients (*n* = 25) received at least one dose of IT nivolumab (Table 1). There was a slight male predominance (*n* = 14, 56%), with a median age of 43 years (range 30–73) at trial entry. Melanoma subtypes included cutaneous (*n* = 10; 40%), primary CNS (*n* = 3; 12%), uveal (*n* = 2; 8%), acral lentiginous (*n* = 1; 4%), mucosal (*n* = 1; 4%) and unknown primary (*n* = 8; 32%). Mutation analysis of tumor tissue revealed BRAF V600E/K mutation in 16 (64%), NRAS mutation in 2 (8%) and GNAQ or GNA11 mutation in 4 (*n* = 2, 8%; uveal melanoma; *n* = 2, 8%, primary CNS melanoma) patients. Most patients had an ECOG performance status of 0 (*n* = 11, 44%) or 1 (*n* = 11, 44%). Extracranial disease was present in 12 (48%) patients and 19 (76%) patients had previous parenchymal MBM. Serum lactate dehydrogenase levels above the institutional upper limit of normal, a well-established prognostic factor in patients with metastatic melanoma, was present in eight patients (32%)^17^.

**Table 1:** Patient demographics and key clinical features

	*n* (%)
Median age at LMD diagnosis (range)	43 (30–73) years
Male	14 (56)
Site of primary melanoma	
Cutaneous	10 (40)
Primary CNS	3 (12)
Uveal	2 (8)
Acral lentiginous	1 (4)
Mucosal	1 (4)
Unknown	8 (32)
Melanoma mutation	
BRAF V600E	15 (60)
BRAF V600K	1 (4)
GNA11/GNAQ	4 (16)
NRAS	2 (8)
Other	2 (8)
Unknown (not sufficient tissue for sequencing)	1 (4)
ECOG performance status	
0	11 (44)
1	11 (44)
2	3 (12)
LDH elevated above institutional limit	8 (32)
CSF positive for malignant cells at baseline	14 (56)
MRI brain findings consistent with LMD at baseline (MRI spine with no evidence of LMD)	12 (48)
MRI spine findings consistent with LMD at baseline (MRI brain with no evidence of LMD)	3 (12)
Both MRI brain and spine consistent with LMD at baseline	10 (40)
Parenchymal brain metastases before LMD diagnosis	19 (76)
Extracranial disease present at time of LMD diagnosis	12 (48)
Prior therapies to LMD diagnosis	
Systemic therapy	23 (92)
Checkpoint inhibitors	21 (84)
BRAF, MEK or BRAF/MEK inhibitors	16 (64)
(Bio)Chemotherapy	3 (12)
Other^a^	5 (20)
Median number of previous metastatic melanoma directed therapies (range)	2 (range 0–6)
Radiation to brain or spine	21 (84)
Whole-brain radiotherapy	9 (36)
Stereotactic radiation	14 (56)
LMD directed systemic therapies before IT/IV nivolumab	8 (32)
Checkpoint inhibitors	3 (12)
BRAF, MEK or BRAF/MEK inhibitors	6 (24)
Chemotherapy	1 (4)
Intrathecal therapy with IL-2	5 (20)
On dexamethasone/corticosteroids at enrollment	10 (40)
Dexamethasone (or equivalent) dose, median (mg d^−1^)^b^	2.0 (range 1–4)
On concurrent BRAF/MEK therapy	11 (44)

LDH, lactate dehydrogenase.

^a^IMCgp100, olaparib, axitinib, relatlimab, azacitadine, TLR9 agonist, CD40 antibody.

^b^Patients requiring corticosteroids.

CSF analysis was done in all patients, and baseline cytopathology was positive in 14 patients (56%). MRI imaging identified findings of LMD in the brain only in 12 patients (48%), in the spine only in 3 patients (12%) and in the brain and spine in 10 patients (40%). Review of systems and physical exam revealed neurological deficits in all patients, albeit subtle in some. Ten (40%) patients were receiving dexamethasone or dexamethasone equivalent (median 2.0 mg d^−1^; range 1–4 mg per 24 h) at initiation of IT nivolumab. For the patients with either history of or concurrent diagnosis of parenchymal brain metastases (*n* = 21), median time from brain metastases to first study treatment was 45.1 weeks (range 3.3–344.3 weeks). Median time from LMD diagnosis to first dose of IT nivolumab was 7.1 weeks (range 0.3–96.4 weeks).

All but two patients (92%) had received previous systemic therapy (median 2, range 1–6). Previous treatments included checkpoint inhibitors (CPI) (*n* = 21, 84%), BRAF/MEK inhibitors (*n* = 16, 64%) and chemotherapy (*n* = 3, 12%). All patients with previous CPI therapy had received anti-PD1 therapy and 14 had received the combination of ipilimumab and nivolumab. Twenty-one patients (84%) had previous radiation to the brain and/or spine, including 12 patients (48%) in whom radiation was directed at the treatment of LMD. Five (20%) patients had received previous IT IL-2.

For patients that developed LMD while on targeted therapy, rapid radiographic and clinical progression was noted in four patients during the initial washout period of 7 d or early during IT nivolumab treatment. After the treatment of 11 total patients, the trial was amended to allow continuation of BRAF/MEK inhibitor therapy. Subsequently, a total of 11 (44%) patients were treated with IT/IV nivolumab with concurrent BRAF/MEK inhibitor combination therapy (IT nivolumab dose 20 mg, *n* = 7; IT nivolumab dose 50 mg, *n* = 4). No other concurrent IT or systemic anti-cancer therapies were permitted.

### Safety

Determination of the safety of IT nivolumab, and of the recommended IT nivolumab dose, were the primary endpoints of the trial. Patients received a median of seven IT nivolumab doses (range, 2–74; Extended Data Table 1). Two patients were treated with 5 mg, three patients with 10 mg (patients were treated in cohorts of two and observed for the 28 d dose-limiting toxicity (DLT) period after the first dose of IT nivolumab). One patient came off protocol before the 28 d DLT period and had to be replaced by the third patient to allow for having two patients monitored for DLT for 28 d, which then would allow for enrollment into the next level. Finally, 14 patients were treated with 20 mg of IT nivolumab, the initial highest planned dose. The 14 patients at the 20 mg of IT nivolumab dose level comprised the 6 patients treated as part of the dose escalation cohort and 8 patients treated on the dose expansion phase before the trial was amended to assess the safety of 50 mg of IT nivolumab (Fig. 1).

IT nivolumab was administered without concurrent IV nivolumab in the first cycle to identify adverse events related exclusively to IT treatment and to allow the assessment of the pharmacodynamic effects of IT administration in the future. Ten patients experienced grade 1 or 2 adverse events at the 5 mg (*n* = 1), 20 mg (*n* = 4) and 50 mg (*n* = 5) of IT nivolumab dose levels. The most frequent adverse events related to IT nivolumab only were nausea (*n* = 7, all grade 1), dizziness (*n* = 4, all grade 1) and vomiting (*n* = 3, all grade 1) (Table 2). One patient experienced neck pain (grade 2). A different patient developed very temporary and self-limiting visual changes, and one patient developed pruritis during cycle 1, whereas another developed self-limiting paresthesia. An additional patient (dose level 5 mg, described in detail below) developed transient and self-limited numbness of the left face (‘paresthesia’, cycle 11) and a 2-min period of transient aphasia (cycle 19) (Table 3). Brain imaging performed to further assess these events did not reveal any concerning findings.

**Table 2:** Adverse events at least possibly related to either IT nivolumab alone or to combined IT and IV nivolumab. After the first cycle (IT nivolumab alone), assignment of toxicity to either IT, IV or IT/IV nivolumab was inherently more difficult because IV and IT nivolumab were administered within 24 h of each other

	IT nivolumab		IT and IV nivolumab	
Adverse event	Grade 1	Grade 2	Grade 1/2	Grade 3
Nausea	7		9	
Diarrhea			6	
Lymphocyte count decreased			5	1
Vomiting	3		3	1
Alanine aminotransferase increased			4	1^b^
Aspartate aminotransferase increased			5	
Headache			4	1
Rash maculopapular			5	
Arthralgia			4	
Dizziness	4		0	
Pruritus	1		4	
Fatigue			2	1
Anemia			2	
Anorexia	1		1	
Dry mouth			2	
Dyspnea			2	
Hypokalemia			2	
Paresthesia	2		0	
Skin and subcutaneous tissue disorders—other, specify			2	
Activated partial thromboplastin time prolonged			1	
Adrenal insufficiency			1	
Alkaline phosphatase increased			1	
Alopecia			1	
Aphasia, transient		1	0	
Blood bilirubin increased			1	
Conjunctivitis			1	
Creatinine increased			1	
Ear pain			1	
Edema limbs			1	
Eye disorders^a^	1		0	
Fever			1	
Gastrointestinal disorders—other, specify			1	
Hyperglycemia			1	
Hypoalbuminemia			1	
Hypocalcemia			1	
Hypoglycemia			1	
Hyponatremia			1	
Hypophosphatemia			1	
Irregular menstruation			0	1
Localized edema			1	
Mucositis oral			1	
Myalgia			1	
Neck pain		1	0	
Neutrophil count decreased			1	
Uveitis			0	1
White blood cell decreased			1	
Total	19	2	84	7

^a^Patient saw prismoid lights after the initial IT nivolumab dose. Self-limiting.

^b^Patient on concurrent therapy with BRAF/MEK inhibitor.

**Table 3:** Breakdown and description of the toxicities of IT nivolumab by IT nivolumab dose level as well as for the individual patient. After the first cycle (IT nivolumab alone), assignment of toxicity to either IT, IV or IT/IV nivolumab was inherently more difficult, as IV and IT nivolumab were administered within 24 h of each other

IT nivolumab dose	Patient Accession Number	Adverse event	Cycle number	Grade 1	Grade 2
5 mg	1	Paresthesia	11	1	
		Aphasia	19		1
20 mg	8	Neck pain	1		1
	10	Nausea	1	1	
	19	Nausea	1	1	
	20^a^	Dizziness	1	1	
		Nausea	1	1	
50 mg	24	Pruritus	1	1	
		Nausea	1	1	
	25^a^	Dizziness	1	1	
		Paresthesia	1	1	
		Vomiting	1	1	
		Nausea	16	1	
	26	Anorexia	1	1	
		Dizziness	1	1	
		Nausea	1	1	
		Vomiting	1	1	
	27^a^	Nausea	1	1	
		Dizziness	1	1	
		Vomiting	1	1	
	30a^a^	Visual changes	1	1	

^a^On concurrent treatment with BRAF/MEK inhibitor.

**Extended Data Fig. 4:**
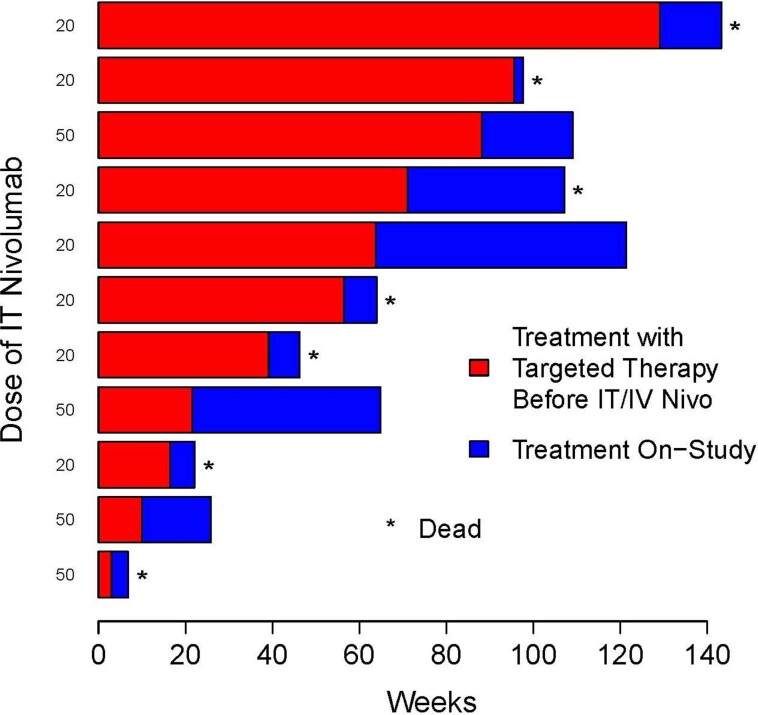
Time on targeted therapy before the first dose of IT nivolumab in BRAF mutant patients who were treated with concurrent BRAF/MEK inhibitors (n = 11). Patients were treated with 20 mg (*n* = 7) and 50 mg (*n* = 4) of IT nivolumab. Only 4 patients remain alive at data lock.

No unexpected toxicities were observed with the addition of IV nivolumab in cycle two. All 25 patients had one or more adverse events related to treatment (Table 2). The most frequently occurring grade 1 or 2 adverse events were nausea (36%), diarrhea (24%), decrease in lymphocyte count (24%), elevation in aspartate aminotransferase and/or alanine aminotransferase (ALT) (24%) and maculopapular rash (24%). Only four patients experienced grade 3 toxicities (total of seven). Of these grade 3 events, one patient developed fatigue, headache and vomiting after two treatment cycles (IT nivolumab dose 50 mg) and was taken off protocol after the third dose owing to rapid disease progression. One additional patient treated at 50 mg experienced a decrease in lymphocyte count (*n* = 1, 4%) after eight cycles of treatment. Two patients (8%, 20 mg of IT nivolumab) developed grade 3 toxicities after 11 cycles (uveitis, *n* = 1, 4%; elevated ALT, *n* = 1, 4%). There was no apparent increase in toxicity observed among patients treated with concurrent targeted therapy (*n* = 11, 44%); and only one grade 3 toxicity (elevated ALT) was observed in a patient treated with concurrent BRAF/MEK inhibitor therapy. Importantly, 8 of 11 patients treated with concurrent targeted therapy had received targeted therapy for at least 5 months before treatment with IT/IV nivolumab (Extended Data Figure 4). Grade 3 and 4 toxicities that were felt to be ‘unlikely’ or ‘unrelated’ to study medication are detailed in the Extended Data Table 2.

At the time of this interim analysis, the study remains open for accrual (planned total enrollment of 50 patients). Because we did not observe any DLTs at the 50 mg of IT nivolumab dose level, all subsequent patients will be enrolled in the 50 mg of IT nivolumab expansion cohort, including patients with LMD from lung cancer.

### Efficacy

**Fig. 2:**
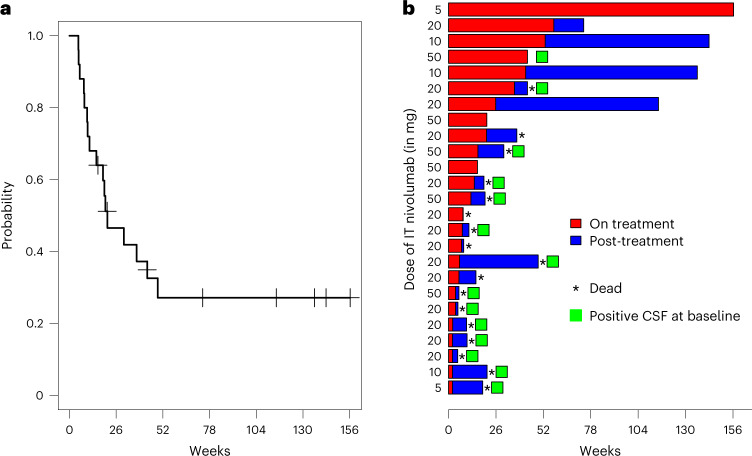
OS and duration on treatment for all treated patients (*n* = 25). **a**, Kaplan–Meier curve of OS for all treated patients. Censored subjects are indicated by tick marks. **b**, Swimmer plot for all treated patients. Bars denote the duration on treatment and time alive after cessation of treatment on clinical trial, the asterisk indicates patients who died and the square indicates patients with positive CSF cytopathology.

**Fig. 3:**
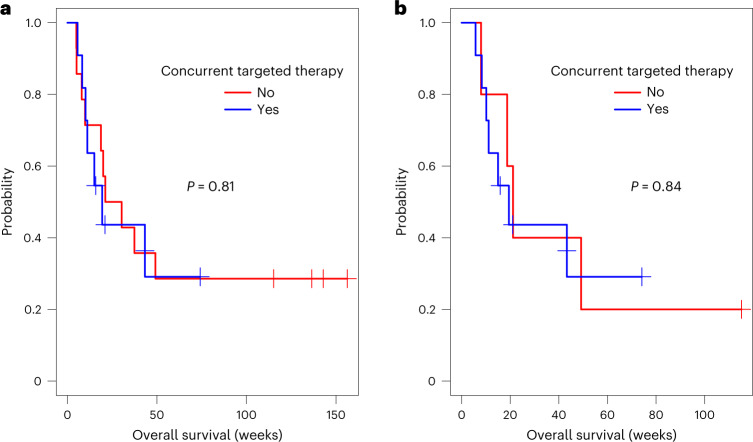
OS for all patients and BRAF mutant patients, stratified by the use of concurrent targeted therapy. **a**,**b**, Kaplan–Meier curves of OS for (**a**) all treated patients (*n* = 25) stratified by the use of concurrent targeted therapy (yes:no = 11:14), regardless of BRAF mutation status and (**b**) BRAF mutant patients (*n* = 16), stratified by the use of concurrent targeted therapy (yes:no = 11:5). Censored subjects are indicated by tick marks. *P* values were derived from the two-sided log-rank test. No adjustment was made for multiple comparisons.

OS was analyzed as a planned secondary endpoint of the trial. At a median follow-up of 20 weeks (range 5–156 weeks), median OS was 4.9 months. OS rates at 13, 26 and 52 weeks were 68%, 44% and 26%, respectively (Fig. 2a). Eight patients were alive at the data lock of 6 June 2021, four of whom continue to receive treatment on trial (Fig. 2b). Among the other surviving patients, three came off study owing to progression and one patient came off owing to requiring therapy for a second, unrelated malignancy (progressive multiple myeloma, which was in remission at time of study enrollment). These four patients remain alive 143, 136, 115 and 74 weeks after the first IT dose, and 94, 90, 89 and 16 weeks after the last IT dose, of nivolumab. Concurrent treatment with targeted therapy (*n* = 11, hazard ratio 1.14, 95% confidence interval (0.324, 4.012), *P* = 0.84) was not associated with a significant difference in OS (Fig. 3a,b). There was also no difference in OS between patients who previously received ipilimumab and nivolumab in combination (*n* = 14) and those who were CPI treatment-naïve (*n* = 4) (hazard ratio 1.31, 95% confidence interval (0.23, 2.81), *P* = 0.68).

**Extended Data Fig. 5:**
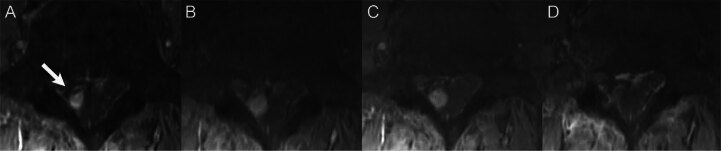
Long term survivor experiencing radiographic complete response. A 57-year-old male treated with IT treatment demonstrates a 9 mm enhancing nodule within the right spinal canal at the L5 level (A) which increases in size to 13 mm at the 4-week follow-up (B) and is stable at 8 weeks (C). Long-term complete response is seen at 3.3 years on the most recent MRI (D). Source data

One patient treated at the initial IT nivolumab dose level of 5 mg has been on treatment for >156 weeks (74 cycles). This patient was diagnosed with BRAF wild-type MBM 4 years before enrollment and was treated with temozolomide, ipilimumab and stereotactic radiosurgery. The patient recurred 3 years later with new parenchymal MBM and was treated again with stereotactic radiosurgery. Five months later, the patient developed lung metastases and was started on systemic nivolumab. Two months after starting nivolumab the patient was found to have LMD on MRI spine imaging. The patient received stereotactic body radiation to the spine, and temozolomide was added to nivolumab treatment. MRI of the spine at 4 and 6 months (at the time of study initiation) after stereotactic body radiation showed worsening LMD, with a concurrent increase in neurological symptoms, including left arm/hand weakness and numbness, neck and back pain. In addition, the patient described occasional dizziness, gait instability and headaches. The patient was enrolled on the clinical trial and received cycle 1 of IT nivolumab 8 months after starting systemic nivolumab treatment. At the time of first dose of IT nivolumab, MRI brain was stable with no radiographic evidence of LMD and computed tomography imaging of the chest, abdomen and pelvis showed mixed response in the lung nodules and increase in size of right liver metastatic disease. CSF analysis at study baseline did not reveal any malignant cells. Initial MRI of the spine performed per protocol 4 weeks after treatment initiation (after two cycles of IT nivolumab) showed worsening radiographic features, but the patient remained clinically stable and decided to continue on protocol treatment. MRI of the CNS and computed tomography imaging of the chest, abdomen and pelvis at 8 weeks showed continuous enlargement of the liver lesion and stable LMD findings in the spine. Because the patient improved clinically (decreased arm numbness and weakness, improved gait and less dizziness), treatment was continued. Subsequent scans have all demonstrated response to treatment, with the most recent scans (36 months after start of treatment) showing ongoing complete radiographic response both on body and CNS imaging (Extended Data Fig. 5), without any significant toxicities. Interestingly, his CSF has been intermittently positive for melanoma cells since cycle 45.

## Discussion

To our knowledge, this trial represents the largest prospective clinical trial of IT immunotherapy in any cancer type and the first to systematically evaluate IT administration of anti-PD1 antibodies^16^. In contrast to the considerable toxicities of IT IL-2, the minimal and manageable toxicities observed with concurrent IT and IV nivolumab demonstrate the feasibility and therapeutic potential of IT administration of CPI-based immunotherapy, and might allow for broader application. Specifically, we have administered the highest planned dose level of 50 mg of IT concurrent with 240 mg of IV nivolumab every 2 weeks safely and consistently for several cycles with most patients stopping treatment secondary to disease progression rather than toxicity. Importantly, we observed preliminary evidence of clinical benefit in some patients previously treated with systemic anti-PD1. Further, these results support the feasibility of conducting prospective clinical trials in melanoma patients with LMD, consistent with recent recommendations by the US Food and Drug Adminstration (FDA) to include patients with CNS disease in clinical trials^18^.

These initial results of our concurrent IT/IV nivolumab trial can be viewed in light of three recently published trials that assessed the efficacy of systemically (IV) administered CPI in patients with LMD, namely pembrolizumab (*n* = 13 and *n* = 20 patients) and the combination of ipilimumab and nivolumab (*n* = 18 patients)^19–21^. In terms of our trial’s primary endpoint of safety, as noted above, we did not observe any new or unexpected toxicities in our trial of IT/IV nivolumab, and mainly observed grade 1 or 2 toxicities. Thus, the described grade 3 and 4 toxicities were even lower than reported in the two trials of IV pembrolizumab (15–40%) in patients with LMD, and lower than the rate reported with IV ipilimumab and nivolumab (33%), despite the addition of IT nivolumab. Notably, IT/IV nivolumab was also much safer than IT IL-2, which results in serious and challenging toxicities in virtually all patients, and thus requires hospitalization^16^.

For the secondary endpoint of OS, the median OS with IT/IV nivolumab was 25 weeks, which is numerically similar to the results observed with IV pembrolizumab (median OS 3.6–4.9 months) and with IV ipilimumab plus nivolumab (median OS 2.9 months). However, a key difference is that, whereas the three previous studies of IV CPI for LMD excluded previous anti-PD1 treatment, the majority (88%) of our cohort had previous exposure to and progression on systemic anti-PD1 (with or without anti-CTLA4). We included anti-PD1-refractory patients in our study because LMD typically represents a late manifestation of disease progression in melanoma, a disease in which CPI is standard frontline therapy. Perhaps consistent with this, the other immune checkpoint inhibitor trials for LMD included very few melanoma patients (two treated with ipilimumab and nivolumab; none treated with pembrolizumab). Breast cancer was the most common diagnosis in those studies (total 22 of 33 patients treated with pembrolizumab; 8 of 18 with ipilimumab and nivolumab), and in retrospective studies has been associated with better outcomes than melanoma in patients with LMD^8,22,23^. The inclusion of anti-PD1-refactory patients allowed us to begin to test the hypothesis that the addition of IT nivolumab to achieve dose intensification in the IT space may overcome CPI resistance in LMD^24^. In support of this, we did observe preliminary evidence of clinical improvement in several anti-PD1-refractory patients. We acknowledge that definitive conclusions about efficacy are not possible from a phase 1 study, but these results, combined with the safety profile, are promising and support the rationale to continue to explore IT/IV nivolumab in LMD.

We did not conclusively observe a clear IT dose–response relationship with either toxicity or efficacy. However, longer follow-up for patients treated with either 20 mg or 50 mg of IT nivolumab is ongoing and we will continue to evaluate a potential relationship between IT dose and clinical benefit, as well as continue monitoring for any long-term toxicity. Planned exploratory analysis of longitudinal CSF samples collected in this study, including cytokine, cell-free DNA and single-cell RNA sequencing analysis, will also assess for dose-dependent effects of IT nivolumab, and/or factors that correlate with clinical outcomes.

As described earlier, concurrent targeted therapy with BRAF or BRAF/MEK inhibitor was initially not permitted. However, after observing several patients with rapid disease progression and clinical deterioration upon targeted therapy discontinuation, an amendment to the protocol allowed continued/concurrent therapy with BRAF/MEK inhibitors, without any dose adjustments. Because concurrent treatment with targeted therapy was not associated with a significant difference in OS when compared with either the entire study population or within the group of BRAF mutant patients, we believe that the promising OS results are still attributable to the IT + IV immunotherapy treatment, but larger patient numbers are needed to confirm this. Reassuringly, among the 11 patients receiving concurrent targeted therapy, none experienced grade ≥3 toxicity with IT nivolumab alone, and only one with concurrent IT/IV nivolumab therapy. Although safety data will continue to be collected in the dose expansion cohort, our current safety results support the feasibility of combining anti-PD1 with targeted therapy in patients with LMD, consistent with the recent approval of vemurafenib, cobimetinib and atezolizumab, as well as ongoing investigations of triplet combination regimens^25^.

In summary, our preliminary analysis demonstrates that concurrent IT and IV administration of nivolumab was safe in melanoma patients with LMD up to the highest planned dose level, with no new or unexpected toxicities, and established the recommended IT nivolumab dose at 50 mg. In this heavily pretreated cohort, there was preliminary evidence of clinical benefit in a subset of patients, including in patients previously treated with systemic anti-PD1. Importantly, our study demonstrates the feasibility of prospective clinical trials for melanoma patients with LMD. These initial results support the rationale for further evaluation of IT anti-PD1 in patients with LMD from melanoma and other cancers.

## Methods

### Study oversight

A copy of the Clinical Protocol is available in the Supplementary Information. The study was designed by the principal investigators and conducted in accordance with the provision of the Declaration of Helsinki and Good Clinical Practice guidelines. University of Texas (UT) MD Anderson Cancer Center (MD Anderson) Institutional Review Board (IRB) approved the protocol after receiving approval for this Investigational New Drug (IND) from the FDA. Study drug and funding was provided by Bristol Myers Squibb (NCT03025256). The study is monitored by the UT MD Anderson IND office and reviewed by the UT MD Anderson Data and Safety Monitoring Committee who evaluate the study on an annual basis.

Study consent was obtained from all study participants before all study-related procedures. No participant compensation was provided.

### Preclinical evaluation of IT anti-PD1

The Institutional Animal Care and Use Committees of MD Anderson approved all mouse experiments. Female only C57BL/6 mice between 8 and 10 weeks old were purchased from the Jackson Laboratory. Experiments using these mice were performed at the MD Anderson South Campus Animal Vivarium. The Department of Veterinary Medicine and Surgery provided veterinary care, including routine husbandry and health monitoring, facilities and services in support of the Institutional Animal Care and Use Program, in keeping with all applicable laws, regulations, guidelines and the Association for Assessment and Accreditation of Laboratory Animal Care accreditation standards. This facility is a dedicated pathogen-free rodent barrier with quarantine and hazard containment provided as well. Mice were kept in HEPA-filtered laminar flow racks in a class 100 cleanroom. Mice were given unlimited access to standard mouse chow and water. Animals are housed in micro-isolators provided by the animal care facility. All animal facilities at MD Anderson operate in compliance with National Institutes of Health regulations as outlined in the DHHS Guide for the Care and Use of Laboratory Animals, the Animal Welfare Act and other relevant Federal Laws. Isotype rat IgG control antibody (BioXCell, clone 2A3, catalog no. BE0089) and anti-mouse PD1 antibody (BioXCell, clone RMP1-14, catalog no. BE0146) were purchased to evaluate the safety of IT anti-PD1 administration.

IT treatments were performed via direct injection into the cisterna magna^26^. The dorsal surface of each mouse was shaved, and mice were anesthetized with an intraperitoneal injection of ketamine/xylazine cocktail (80 mg per kg (body weight) ketamine and 12 mg per kg (body weight) xylazine). Percutaneous IT injection was performed with a 30-gauge Hamilton needle^27^. To perform the injection, mice were placed in the prone position with the neck flexed over a 15 ml conical centrifuge tube. The head was immobilized with the thumb and index finger. The space between the occiput and the C1 vertebrae was palpated, and the entry point was marked on the skin. The needle was marked at a depth of 4 mm, and the animal was injected at an approximately 45° angle. Solution was dispensed slowly over 2 min into the cisterna magna, and the needle was then withdrawn. The mice were then resuscitated from anesthesia. To ensure the viability of this technique and confirm the distribution of injected material to the subarachnoid space/leptomeninges, we injected 10 μl of Evans (methylene) blue dye intrathecally into C57BL/6 mice. Following IT injection, mice were killed. Brain and spinal cord tissue were removed and grossly examined.

### IT delivery of anti-PD1 in nontumor-bearing mice

The safety of IT administration of anti-PD1 antibody was evaluated in nontumor-bearing immunocompetent mice. The dose of IT anti-PD1 used in the experiments was determined two ways. The first derivation was based on the use of a dose in mice that was equivalent by volume of CSF to 50 mg of IT anti-PD1 in humans, because 50 mg is a dose used with IT trastuzumab and IT rituximab, and in preliminary discussions was considered the maximum dose of IT nivolumab to be evaluated in the clinical trial. Based on differences in CSF volume of distribution, we determined that a dose of 6.5 μl (13 μg) of anti-PD1 would be equivalent to a dose of 50 mg of IT nivolumab in patients. The second approach was based on the differences in CSF volume of distribution between humans and mice (140 ml versus 36 µl), we determined that a dose of 13 µg of anti-PD1 in mice would be equivalent to a dose of 50 mg in patients. Murine IT dose based on this calculation was 6.5 μl of murine anti-PD1 concentration of 2,000 µl ml^−1^, and therefore, the highest volume (6.5 μl) for injection was chosen.

Sixty-eight female only C57BL/6 mice were divided into three volume-matched (6.5 μl) treatment groups: (1) IT PBS control; (2) IT isotype control (13 µg; rat IgG2a; BioXCell, clone 2A3); and 3) IT mouse anti-PD1 antibody (13 μg; BioXCell, clone RMP1-14). Mice were killed at 48 h, 7 d and 14 d after IT injection. Brain and spinal cord tissue were collected from mice at all three time points for histological assessment for neurotoxicity. Brain regions and spinal cords were embedded in paraffin and sectioned at 5 µm and stained with H&E for histological observation. All IHC studies were performed on 5 µm sections. Samples were assessed for known inflammatory markers by IHC, including CD3 (SP7; Thermo Fisher, catalog no. RM-9107-S, dilution 1:100), CD8 (D4W2Z; Cell Signaling, catalog no. 98941, dilution 1:400), CD163 (polyclonal; ProteinTech, catalog no. 16646-1-A, dilution 1:200), CD56 (polyclonal; ProteinTech, catalog no. 14255-1-AP, dilution 1:3,000), CD15 (FUT4/1478R; Abcam, catalog no. ab218403, dilution 1:20), PD1 (D7D5W; Cell Signaling, catalog no. 84651, dilution 1:100) and PD-L1 (D5V3B; Cell Signaling, catalog no. 64988, dilution 1:100).

### Study design, treatment and endpoints

This study is an open-label, single-center, phase 1/1b first-in-human dose escalation and expansion clinical trial using concurrent IT and IV nivolumab. The study consisted of two parts: a phase 1 dose escalation portion and a phase 1b dose expansion cohort at the recommended dose. Each cycle was 14 d. Cycle 1 included treatment with IT nivolumab only, subsequent cycles included IT and IV nivolumab treatments. All IT doses were administered via an Ommaya reservoir. Initial IT nivolumab dose levels were flat doses of 5, 10 and 20 mg administered over 5 min. No prophylactic medication was given, but patients were monitored in the hospital for 24 h after each IT nivolumab dose.

The initial version of this protocol allowed for patients to be dose escalated up to 20 mg of IT nivolumab. Because this was a first-in-human IT administration of this treatment, safety was routinely evaluated in accordance with our data safety plan. Because no concerning safety signals were noted, the decision to escalate to 50 mg of IT nivolumab (dose level 4, administered over 5 min) in pursuit of a MTD was discussed with the MD Anderson IND office as well as Bristol Myers Squibb and reviewed and approved by the FDA. Importantly, during the initial murine experiments, IT murine anti-PD1 doses equivalent to this human dose were tested and found to be safe.

Once the maximum delivered dose was deemed safe, according to the operating rules of the statistical design, the study proceeded with the expansion cohort at the recommended phase 1b dose (Fig. 1).

Intrapatient dose escalation was not permitted. IV nivolumab was administered at a flat dose of 240 mg over 30 min every two weeks 24 h after the IT nivolumab dose, regardless of the IT dose level. Patients were enrolled in cohorts of two; to ensure maximum safety for subsequent patients, IT nivolumab start dates were staggered between each subsequent patient with a minimum of 48 h at each dose level.

The primary endpoint was to determine the safety and recommended dose of IT nivolumab in combination with IV nivolumab treatment in patients with LMD. OS was a secondary objective, and exploratory objectives included clinical, molecular and immune predictors of safety and efficacy of the regimen, and evaluation of the levels of nivolumab in the CSF and peripheral blood after IT administration.

Disease response assessment consisted of cytopathological analysis of CSF every 2 weeks for the initial 8 weeks, and every 8 weeks thereafter. CNS imaging was performed at baseline; at 4 and at 8 weeks; and every 8 weeks thereafter. All CNS imaging was reviewed by two board-certified neuroradiologists.

### Statistical analysis

The Bayesian modified toxicity probability interval method of Ji et al. was used to find the MTD of IT dose of nivolumab^28^. DLTs of IT nivolumab were recorded. DLTs were defined as the development of grade 3 or higher CNS toxicity or systemic toxicity. The timeline for assessing DLTs was for 28 d after the initiation of IT nivolumab (day 1, cycle 1). The targeted DLT rate was 30%, and the targeted DLT interval by modified toxicity probability interval was (25%, 35%). We assumed a previous Beta(1,1) distribution for the DLT rate at each dose level. After dose escalation was complete, the MTD was defined as the highest dose for which the posterior estimate of the DLT rate is closest to 30%. Once the recommended dose of nivolumab was established, an additional eight patients were treated at this dose in a phase 1b expansion cohort. In addition, the protocol was modified to enroll patients at a higher IT nivolumab dose of 50 mg.

For the secondary objective, the Kaplan–Meier method was used to estimate the distribution of OS from the start of study treatment. Cox proportional hazards regression was used to assess the relationship between OS and various covariates of interest, including but not limited to patient demographics, tumor characteristics and disease characteristics. All statistical tests were performed two-sided, and no adjustment was made for multiple testing.

## Online content

Any methods, additional references, Nature Portfolio reporting summaries, source data, extended data, supplementary information, acknowledgements, peer review information; details of author contributions and competing interests; and statements of data and code availability are available at 10.1038/s41591-022-02170-x.

### Supplementary information

Supplementary Information Clinical Protocol.

### Source data

Source Data Extended Data Fig. 2 Source Data for Extended Data Fig. 2, Excel.

## Figures and Tables

**Extended Data Table 1:**
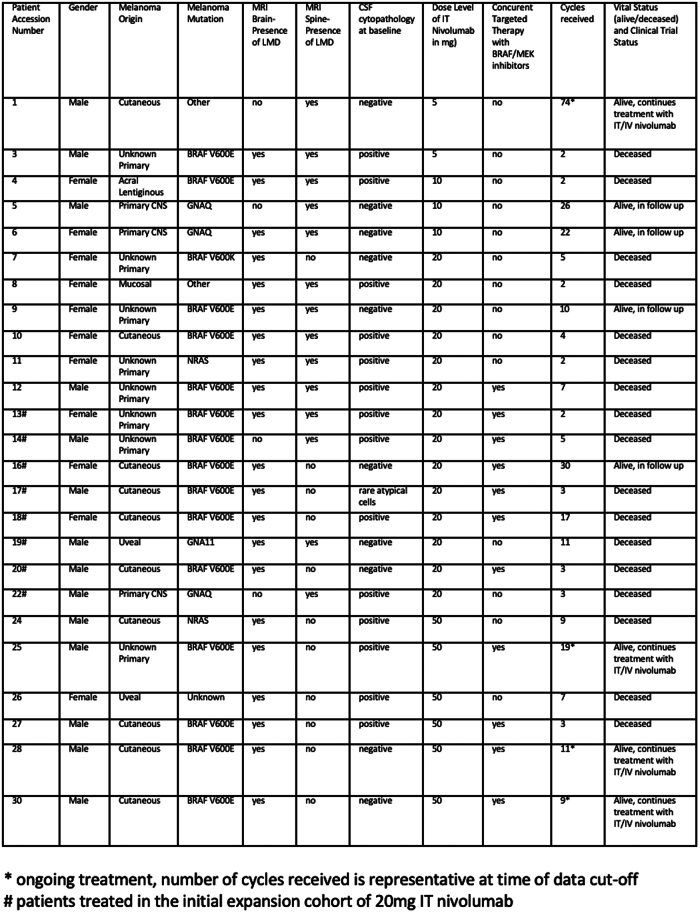
Overview of pertinent patient characteristics, as well as BRAF status and total cycles of IT nivolumab received by dose level, patient accession number and outcome of patients

**Extended Data Table 2:**
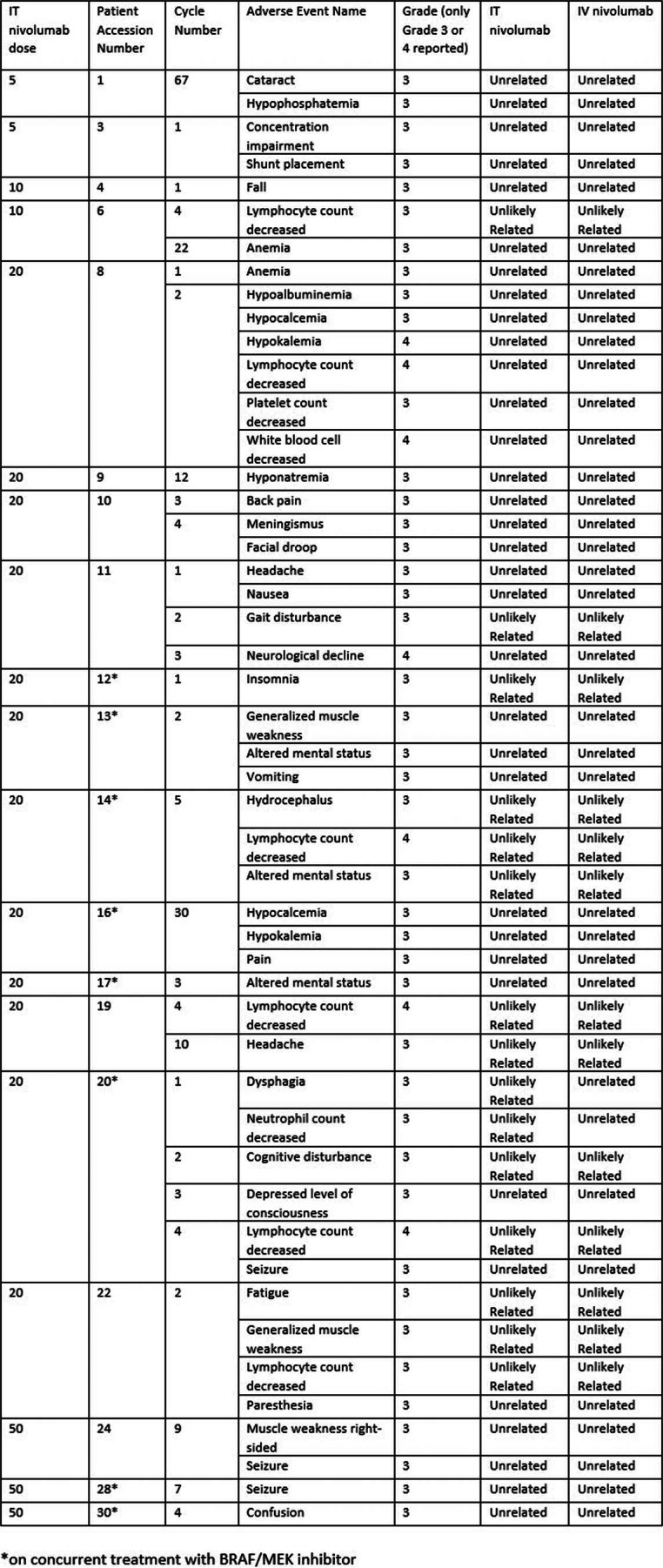
Breakdown and description of grade 3 and 4 toxicities, assigned as ‘unlikely related or unrelated to study medication’ medication, by IT nivolumab dose level and individual patient

## Data Availability

Patient-related data not included in the paper were generated as part of a clinical trial and are subject to patient confidentiality, and de-identified clinical data can be made available with the reporting of the final clinical outcomes of the study. The full study protocol is available in the Supplementary Information. Any requests for additional clinical data will be reviewed by the University of Texas MD Anderson Institutional Review Board (IRB). Any data and materials (for example, tissue samples or imaging data) that can be shared will need approval from the UT MD Anderson IRB and a Material Transfer Agreement in place. Source data for Extended Data Fig. 2 are available. Any requests for clinical data should be addressed to the corresponding author Isabella C. Glitza Oliva (icglitza@mdanderson.org).
