# Author Correction: Concurrent intrathecal and intravenous nivolumab in leptomeningeal disease: phase 1 trial interim results

**DOI:** 10.1038/s41591-024-02998-5

**Published:** 2024-04-22

**Authors:** Isabella C. Glitza Oliva, Sherise D. Ferguson, Roland Bassett, Alexandra P. Foster, Ida John, Tarin D. Hennegan, Michelle Rohlfs, Jessie Richard, Masood Iqbal, Tina Dett, Carol Lacey, Natalie Jackson, Theresa Rodgers, Suzanne Phillips, Sheila Duncan, Lauren Haydu, Ruitao Lin, Rodabe N. Amaria, Michael K. Wong, Adi Diab, Cassian Yee, Sapna P. Patel, Jennifer L. McQuade, Grant M. Fischer, Ian E. McCutcheon, Barbara J. O’Brien, Sudhakar Tummala, Matthew Debnam, Nandita Guha-Thakurta, Jennifer A. Wargo, Fernando C. L. Carapeto, Courtney W. Hudgens, Jason T. Huse, Michael T. Tetzlaff, Elizabeth M. Burton, Hussein A. Tawbi, Michael A. Davies

**Affiliations:** 1 Department of Melanoma Medical Oncology, The University of Texas MD Anderson Cancer Center, Houston, TX USA; 2 Department of Neurosurgery, The University of Texas MD Anderson Cancer Center, Houston, TX USA; 3 Department of Biostatistics, The University of Texas MD Anderson Cancer Center, Houston, TX USA; 4 Department of Surgical Oncology, The University of Texas MD Anderson Cancer Center, Houston, TX USA; 5 Department of Neuro-Oncology, The University of Texas MD Anderson Cancer Center, Houston, TX USA; 6 Department of Neuroradiology, The University of Texas MD Anderson Cancer Center, Houston, TX USA; 7 Department of Translational Molecular Pathology, The University of Texas MD Anderson Cancer Center, Houston, TX USA; 8 Department of Pathology, The University of Texas MD Anderson Cancer Center, Houston, TX USA; 9 Department of Pathology, University of California San Francisco, San Francisco, CA USA; 10 Department of Genomic Medicine, The University of Texas MD Anderson Cancer Center, Houston, TX USA

**Keywords:** Cancer immunotherapy, Phase I trials, CNS cancer, Melanoma

Correction to: *Nature Medicine* 10.1038/s41591-022-02170-x, published online 30 March 2023.

In the version of the article initially published, Fernando C. L. Carapeto (Department of Translational Molecular Pathology, The University of Texas MD Anderson Cancer Center, Houston, TX, USA), Courtney W. Hudgens (Department of Translational Molecular Pathology, The University of Texas MD Anderson Cancer Center, Houston, TX, USA), Jason T. Huse (Department of Pathology, The University of Texas MD Anderson Cancer Center, Houston, TX, USA) and Michael T. Tetzlaff (Department of Pathology, University of California San Francisco, San Francisco, CA, USA) were mistakenly omitted from the author list and contributions (performed the pathology and associated figures) and have now been added in the HTML and PDF versions of the article.

