# Supplementary material for: Concurrent intrathecal and intravenous nivolumab in leptomeningeal disease: phase 1 trial interim results
Source: Nat Med. 2023 Mar 30;29(4):898–905. doi: 10.1038/s41591-022-02170-x (PMC10115650; doi:10.1038/s41591-022-02170-x)
Supplement: Supplementary file 1 — Clinical Protocol. [file 41591_2022_2170_MOESM1_ESM.pdf]

nature medicine

Article
https://doi.org/10.1038/s41591-022-02170-x

Concurrent intrathecal and intravenous nivolumab in leptomeningeal disease: phase 1 trial interim results

In the format provided by the authors and unedited

MD Anderson IND Sponsor Cover Sheet	
**Protocol ID**	2016-0567
**Protocol Title**	**Phase I/Ib study of concurrent intravenous and intrathecal nivolumab for melanoma and lung cancer patients with leptomeningeal disease (LMD)**
**Protocol Phase**	I/Ib
**Protocol Version**	8
**Version Date**	July 9, 2020
**Protocol PI**	Isabella Claudia Glitza, MD, PhD
**Department**	Melanoma Medical Oncology
**Investigational Products**	**BMS-936558 (nivolumab)**
**IND Sponsor**	MD Anderson Cancer Center
**IND #**	133201

Phase I/Ib study of concurrent intravenous and intrathecal nivolumab for melanoma and and lung cancer patients with leptomeningeal disease (LMD)

Principal Investigator: Isabella Claudia Glitza, MD, PhD

Co-Principal Investigator: Michael A. Davies, MD, PhD

Departments of Melanoma Medical Oncology

The University of Texas MD Anderson Cancer Center

BMS Study No.: CA209-699

Table of Contents

Contents

Table of Contents 3
1. SYNOPSIS 6
OBJECTIVES: 7
STUDY ASSESSMENTS 8
STATISTICAL CONSIDERATIONS 9
BACKGROUND AND RATIONALE 9
1.1. Introduction 9
1.2. Nivolumab 10
1.3. Experience with intrathecal therapy approach 12
1.3.1. Ommaya 12
1.4. Preliminary Safety Results of clinical trial 13
2. OBJECTIVES AND ENDPOINTS 13
2.1. Primary Objective 13
2.2. Secondary Objectives 13
2.3. Exploratory Objectives 13
3. ETHICAL CONSIDERATIONS 14
3.1. Good Clinical Practice 14
3.2. Institutional Review Board/Independent Ethics Committee 14
3.3. Informed Consent 14
Investigators must: 15
4. INVESTIGATIONAL PLAN 16
4.1. Study Design 16
4.2. Eligibility Criteria 16
4.2.1. Inclusion Criteria 16
Table 1. Eligibility Guidelines of Organ and Marrow Function 18
4.2.2. Exclusion Criteria 22
4.3. Study Design Schema 24
4.4. Treatment Assignment 25
4.5. Investigational Product 25
4.6. Intrathecal Nivolumab 25

4.6.2. Dose Escalation and Administration of Intrathecal Nivolumab ... 25
Table 2: Dose escalation for intrathecal and intravenous nivolumab ... 26
4.6.2. Dose levels ... 27
4.6.3. Administration and Timing of IT nivolumab ... 28
4.6.4. Dose Modifications ... 30
4.7. Intravenous Nivolumab ... 30
4.7.1. Dose Modifications ... 31
4.8. Dose Delay Criteria ... 31
4.9. Intrathecal Dose Delay ... 31
4.10. Systemic Dose Delay ... 31
4.11. Criteria to Resume Treatment ... 32
4.11.1. Intrathecal Dose ... 32
4.11.2. Systemic Dose ... 32
4.11.3. Discontinuation Criteria ... 33
4.11.4. Treatment of Nivolumab Related Infusion Reactions ... 36
4.11.5. Drug ordering and accountability ... 38
4.11.6. Handling and Dispensing ... 39
4.11.7. Destruction ... 39
4.12. Product Descriptions ... 40
Table 4: Product description ... 41
4.12.1. Study Design: Dose expansion cohort ... 42
4.12.2. Permanent Discontinuation from Study Treatment and Subject Completion Criteria ... 42
4.12.3. Prohibited Medications and Non-drug Therapies ... 44
4.12.4. Treatment Beyond Progression ... 44
4.12.5. Isolated systemic disease progression ... 46
5. SCHEDULE OF ASSESSMENTS ... 47
5.1 Study Procedure Tables ... 47
Table 5: Screening, labs/biopsies, Imaging and Diagnostic tests at baseline and on study ... 47
Table 6: Nivolumab dosing, ICP monitoring, CSF and blood sampling ... 50
5.2 Critical Baseline Assessments ... 51
5.3 Safety Evaluations on Therapy ... 52
5.3.1 Physical Exams/ECOG/Vital Signs/Concomitant Medications ... 52

5.3.2 Adverse Event Assessment ... 52
5.3.3 Laboratory assessments ... 52
Hematology ... 53
5.4 Biospecimen collection ... 53
5.4.1 CSF collection, and intracranial pressure monitoring ... 54
5.4.2 Peripheral Blood and Serum Samples ... 56
5.4.3 Tumor Biopsies and Surgical Specimen ... 56
5.5 Disease Response Assessments ... 57
5.5.2. Imaging Assessments ... 57
5.5.3. Neurological Exam and Neurocognitive function test ... 60
Table 10: Neurological examination key points ... 61
5.5.4. Definition of Response ... 63
5.5.5. Translational Research/Biomarker Analysis ... 64
6 SAFETY AND REPORTING ... 65
6.1 Serious Adverse Event Reporting (SAE) Language ... 65
6.2 Reporting to FDA: ... 66
6.3 Investigator Communication with Supporting Company BMS: ... 67
6.3.1 SAE Reporting ... 67
6.3.2 Serious Adverse Event Collection and Reporting ... 69
6.3.3 Non-serious Adverse Event Collection and Reporting ... 72
6.3.4 Laboratory Test Abnormalities ... 72
6.3.5 Pregnancy ... 72
6.3.6 Overdose ... 73
6.3.7 Other Safety Considerations ... 73
7 STATISTICAL CONSIDERATIONS ... 73
7.1 Dose Escalation Part ... 74
Figure 4 75
7.2 Dose Expansion ... 81
7.3 Patient Characteristics ... 86
7.4 Exploratory Efficacy Measures ... 86
7.5 Safety Measures ... 87
8 STUDY MANAGEMENT ... 88

8.1 Data Management 88
8.2 Posting of Information on Clinicaltrials.gov 88
8.3 Records Retention 88
9 REFERENCES 89
10. APPENDICES 91
10.1. RECIST 1.1 Criteria Measurability of tumor lesions at baseline Measurable lesion: 91
Non-measurable lesion: 91
Measurable disease: 92
Non-Measurable only disease: 92
Specifications by methods of measurements 92
Clinical Examination: 92
Contrast enhanced CT with 5mm contiguous slices is recommended. 93
Evaluation of target lesions: 93
Note: 94
Evaluation of non-target lesions 94
Note: 95
Confirmation of response 95
Overall response criteria 95
Note: 96
UNACCEPTABLE METHODS OF CONTRACEPTION 98

1. SYNOPSIS

Collaborators:

Medical Oncology:	Neurosurgery:	Neurooncology:
Hussein Tawbi, MD<br>Rodabe Amaria, MD<br>Adi Diab, MD<br>Patrick Hwu, MD<br>Sapna Patel, MD<br>Scott Woodman, MD, PhD<br>Cassian Yee, MD. PhD	Ian McCutcheon, MD<br>Sherise Ferguson, MD<br><br>**Biostatistics:**<br>Roland Bassett Jr.<br><br>**Neuroradiology:**<br>Nandita Guha-Thakurta, MD	Barbara O'Brian, MD<br><br>**Pathology:**<br>Alexander Lazar, MD, PhD<br>Michael Tetzlaff, MD, PhD

STUDY SYNOPSIS

Background Information

Metastasis to the central nervous system (CNS) remains one of the most common and devastating complications of advanced melanoma. Up to 60% of metastatic melanoma patients are clinically diagnosed with CNS disease, and this immune-privileged site is a frequent site of treatment failure among patients treated with approved therapies. Our previous review of outcomes of advanced melanoma patients demonstrated that the presence of leptomeningeal disease (LMD) predicts the worst outcomes among patients with CNS metastases (median survival 1.8 months from diagnosis).[1] Treatment options for patients with LMD are very limited, and there is minimal evidence of clinical benefit from them. Our unique and long- term experience with intrathecal interleukin-2 (IT IL2) therapy has demonstrated that intrathecal immunotherapy can achieve durable disease control and survival in patients with LMD. As systemic administration of nivolumab, an anti-PD-1 antibody, has favorable clinical activity and safety compared to systemic IL2 therapy, there is a strong rationale to determine the safety and efficacy of intrathecal nivolumab in melanoma patients with LMD.

Investigational Product(s), Dose and Mode of Administration, Duration of Treatment with Investigational Product(s)

The clinical trial will consist of two parts:

(1) intrathecal dose-escalation portion with 4 dose levels (5, 10,-20, 50 mg of intrathecal nivolumab)

(2) dose-expansion cohort at the recommended Phase Ib dose. Each treatment cycle will be 14 days.

Nivolumab (BMS-936558) administered IV over 30 minutes at 240 mg every 2 weeks ongoing.

Nivolumab (BMS-936558) administered intrathecally at4 different dose levels (see Table 2) over 5 minutes every 2 weeks.

Study Phase: I/Ib

Research Hypothesis: The central hypothesis is that IT administration of nivolumab is safe and can induce immune responses in the CSF in patients with LMD

OBJECTIVES:

Primary Objective

The primary objective of this study is to determine the safety and/or recommended dose of intrathecal (IT) nivolumab in combination with systemic nivolumab treatment in patients with LMD.

Secondary Objectives

1. To assess overall survival with combined intrathecal and systemic administration of nivolumab in this patient population.

Exploratory Objectives

1. Compare the immunological effects of this treatment on immune cells in the CSF to those observed in the peripheral blood and in non-LMD tumors.
2. Evaluation of predictors (clinical, molecular, and/or immune) of the efficacy and safety of this regimen.
3. To assess the effect of nivolumab on subsequent treatment.
4. To compare levels of nivolumab in the CSF and peripheral blood

STUDY DESIGN

This is a Phase I/Ib, non- randomized trial of concurrent intravenous and intrathecal nivolumab in adult (≥18 years) patients with leptomeningeal disease from melanoma or lung cancer. Patients must have radiographic and/or CSF cytological evidence of LMD. Patients with prior therapy with α-PD-1

and/or anti-CTLA-4 will be eligible.

This clinical trial will consist of two parts: (1) a dose-escalation portion, and (2) a dose-expansion cohort at the recommended Phase Ib dose.

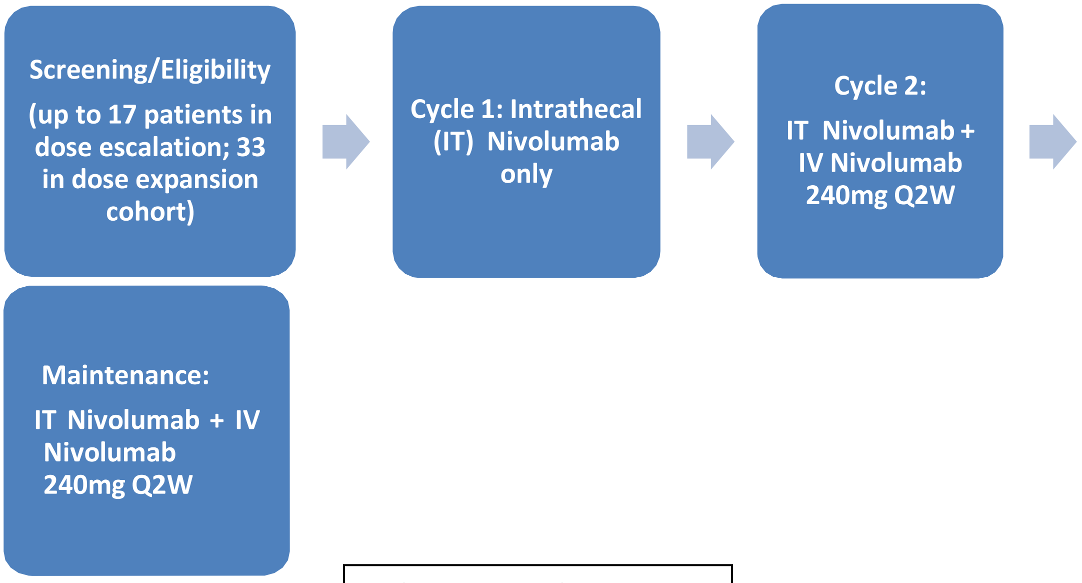

Cycle 1: Dose escalation,
Dose level -1: 1mg flat dose
Dose level 1: 5mg flat dose
Dose level 2: 10 mg flat dose
Dose level 3: 20 mg flat dose.
Dose level 4: 50mg flat dose

STUDY POPULATION

This study will enroll patients with histologically confirmed metastatic melanoma and lung cancer presenting with confirmed leptomeningeal disease.

STUDY ASSESSMENTS

The primary objective of this study is to determine the safety and recommended dose of intrathecal (IT) nivolumab in combination with systemic nivolumab treatment in patients with

LMD.

Safety will be evaluated for all treated subjects using the NCI CTCAE version 4.03. Safety assessments will be based on medical review of AE reports and the results of vital sign measurements, physical examinations, and clinical laboratory tests. Clinical decisions to continue or hold/discontinue therapy will be based on the judgment of the treating physician/principal investigator.

Exploratory biomarker assessments are included to evaluate associations with clinical endpoints, safety and tolerability, as well as for the identification of potential predictive markers for future studies or practice.

STATISTICAL CONSIDERATIONS

Sample Size: 17 to 50 patients (up to 17 patients in dose escalation; 33 in dose expansion cohort).

BACKGROUND AND RATIONALE

1.1. Introduction

The spread of cancer to the central nervous system (CNS) is a devastating complication of multiple tumor types, particularly melanoma, lung, and breast cancer. While there are effective treatment options available for patients with extracranial metastatic disease that may translate into benefit for parenchymal brain metastases, there are very few options for patients with leptomeningeal disease (LMD) from any cancer type. The current primary treatments for LMD are palliative radiation therapy (RT), steroids and/or supportive care for related symptoms. As LMD is also a frequent site of disease progression in patients treated with systemic therapies, and as the diagnosis of LMD is associated with extremely poor prognosis (i.e. median overall survival [OS] of 1.8 months from diagnosis for melanoma patients with evidence of LMD),[1] the development of effective therapy approaches for LMD could have tremendous clinical impact. Nivolumab has demonstrated impressive efficacy and safety in melanoma.[2] We hypothesize that increasing the exposure to nivolumab in the CSF by direct IT administration will be feasible, safe, and will positively impact disease control and survival in metastatic melanoma patients with LMD.

Based on the favorable side effect profile and efficacy of nivolumab compared to systemic IL-2, we

hypothesize that IT administration of nivolumab will be a safe and effective treatment for metastatic melanoma patients with LMD. We also believe that the safety profile of systemic nivolumab will allow for concurrent IV administration, which is important for the treatment of co-existing extracranial disease in these patients. Thus, we will conduct a single-center phase I/Ib clinical trial to determine the safety, efficacy, and immunological effects of IT administration of nivolumab in combination with systemic nivolumab treatment in metastatic melanoma patients with LMD. The trial will consist of an initial IT dose-finding phase to determine the tolerance of IT nivolumab. CSF will be collected to analyze the pharmacokinetics of nivolumab in the CSF, and levels will be compared to those achieved in the blood. Exploratory studies will also evaluate the immunological effects of this regimen in the CSF, the blood, and safely accessible non-CNS tumors; predictors of clinical efficacy and toxicity will also be explored.

Dose escalation and final dose selection will most appropriately be determined based on combined evaluation of safety and exposure, followed by a dose expansion cohort to further define the safety and assess the efficacy of the selected dose. It is conceivable that, upon determination of a recommended dose for further testing, additional disease-specific dose-expansion cohorts could be considered in patients with LMD from other cancers such as lung cancer, and breast cancer.

1.2. Nivolumab

Programmed death 1 (PD-1) is a more recently discovered immune checkpoint receptor that has generated considerable excitement based on favorable preclinical profiling and initial clinical results. Like **CTLA**-4, PD-1 is a transmembrane protein expressed on effector immune cells. [3] Also like **CTLA**-4, expression of PD-1 is inducibly expressed with lymphocyte activation, although it is expressed more broadly than CTLA-4 as it is also found on activated B lymphocytes and natural killer cells. [4,5-] PD-1 is bound principally by programmed death ligand 1 (PD-L1, B7-H1) but also, to a lesser degree, by programmed death ligand 2 (PD-L2, B7-DC).[6]

The PD-1 receptor pathway is an important negative regulator of the immune system. The PD-1 pathway appears to also have a primary role in cancer tolerance and immune escape. Higher expression of PD-L1 on tumor cells is associated with a worse prognosis, more aggressive features, and/or resistance to immunotherapy in the large majority of cancers that it has been characterized

in. [7] Furthermore, preclinical data demonstrates that increasing tumor expression of PD-L1 makes it less susceptible to immunotherapy, while blocking it increases its vulnerability to immune-mediated destruction.[8]

Based on promising preclinical therapeutic results, PD-1 blocking mAbs have proceeded to human clinical trials. [8-10] Nivolumab (MDX-1106, BMS-936558, Bristol-Myers Squibb, New York, NY) is a fully humanized IgG4 monocolonal antibody that binds to PD-1, blocking its binding site. It was initially tested in a phase I, dose escalation trial in 296 patients with heavily pretreated advanced melanoma (n=104), colorectal cancer (n=19), CRPC (n=17), NSCLC (n=122), and renal cell carcinoma (n=34). Nivolumab was given at 0.3, 1, 3, or 10 mg/kg in six patient cohorts followed by expansion cohorts at 10mg/kg. Patients were initially given a single dose and allowed additional doses if they demonstrated clinical benefit, however, the trial transitioned into a phase Ib where patients were dosed every two weeks and reassessed every eight weeks. Treatment was continued for up to 96 weeks or until disease progression or complete response. Treatment with nivolumab was better tolerated overall than treatment with CTLA-4 blocking antibodies with no maximum tolerated dose achieved. Only 14% experienced serious (≥ grade 3) drug toxicity and only 5% had to discontinue therapy secondary to this.

Nivolumab treatment demonstrated substantial antitumor effect, with partial or complete responses (by RECIST criteria) observed in patients with melanoma, NSCLC, and renal cell carcinoma but not colorectal cancer or CRPC. Responses were observed across various doses at rates of 19 to 41% in melanoma, 6 to 32% in NSCLC, and 24 to 31% in renal cell carcinoma. In addition, disease stability and mixed response were observed in a substantial portion of patients. In melanoma patients two and three year OS were 48% and 41%, respectively and the median duration of response in patients with an objective response was an impressive 22.9 months.[11] Further analysis of PD-L1 expression from 61 patients who had pretreatment specimens available demonstrated an objective response in 36% of tumors expressing PD-L1 and none in PD-L1-negative tumors.[17]

Recently, the FDA recommend dose was changed from 3mg/kg to flat dose of 240mg IV over 60 minutes every 2 weeks. This was based on dose/efficacy and safety relationships, and there was no clinical significant difference in safety and efficacy of nivolumab administered every 2 weeks at a

flat dose of 240mg versus 3mg/kg in patients with metastatic melanoma. No premedication is required.

1.3. Experience with intrathecal therapy approach

Previous experience with trastuzumab (in breast cancer) and rituximab (in lymphoma) support that intrathecal (IT) administration of therapeutic antibodies may be required to achieve significant drug exposure in the CSF. [12-15] Those non-randomized studies also provide proof-of-principle that IT administration of antibodies can be safe and achieve clinical benefit.[15]

The Department of Melanoma Medical Oncology at the MD Anderson Cancer Center (MDACC) includes a unique and long-standing intrathecal IL-2 (IT IL2) program.[16] We have treated 100 melanoma patients with IT IL2 over the last 20 years. The analysis of a contemporary series of 43consecutive melanoma patients treated with IT IL2 at our center between 2006 and 2014 showed 1-, 2-, and 5-year survival rates of 36%, 26% and 13%, respectively from the start of treatment.

While these results provide compelling evidence that IT immunotherapy can achieve long-term survival, there is a critical need to identify therapies that are more effective. In addition, IT IL2 treatment causes significant toxicities in virtually all patients due to increased intracranial pressure (ICP), and thus requires specialized care and prolonged hospitalization for patient safety and symptom control. Notably, as part of this program we have developed a large team of mid-level providers that can provide the specialized care for patients with LMD in both the inpatient and outpatient settings, including the administration of IT immunotherapy and the recognition and management of side effects, including CSF removal. This unique infrastructure, along with established SOPs to collect and process CSF for research, allows us to evaluate the safety, efficacy, and immunological effects of IT immunotherapy for LMD.

1.3.1. Ommaya

1.3.1.1. Placement of Ommaya

All patients require the presence of an Ommaya reservoir prior to the first dose if IT nivolumab. Ommaya reservoirs are standard of care in the management of patients with LMD.

1.3.1.2. Safety Consideration of Ommaya

Only specially trained personnel that followed standardized guidelines will access the reservoir and administer the drug. In general, complications are very rare and can be separated into 2 categories: early complication due to placement, and delayed complication due to infection, or change in position of the catheter. All of these are exceedingly rare. [17-19]

1.4. Preliminary Safety Results of clinical trial

As of March 2020, 15 patients have been treated to date on clinical trial with IT Nivolumab.
During the dose escalation, the treatment was as following: Two patients were treated at Dose level 1 (5mg) of IT Nivolumab, 3 patients were treated at Dose level 2 (10mg) of IT Nivolumab, as one patient did come off trial due to disease progression before the end of the 28 day DLT period, and 6 patients were treated at Dose Level 3 (20mg) of IT Nivolumab. The MD Anderson IRB required to have a total of 6 patients treated at Dose Level 3 without any DLTs before opening the expansion cohort.
In the dose expansion, 5 patients have been treated thus far at the IT nivolumab dose level of 20mg. To date, we have not experienced any DLT related to the IT nivolumab administration.

2. OBJECTIVES AND ENDPOINTS

2.1. Primary Objective

- The primary objective of this study is to determine the safety and recommended dose of intrathecal (IT) nivolumab in combination with systemic nivolumab treatment in melanoma and lung cancer patients with LMD.

2.2. Secondary Objectives

- To assess overall survival with combined intrathecal and systemic administration of nivolumab in this patient population.

2.3. Exploratory Objectives

- Compare the immunological effects of this treatment on immune cells in the CSF to those observed in the peripheral blood and in non-LMD tumors.
- Evaluation of predictors (clinical, molecular, and/or immune) of the efficacy and safety of this regimen.
- To assess the effect of nivolumab on subsequent treatment.
- To compare levels of nivolumab in the CSF and peripheral blood

3. ETHICAL CONSIDERATIONS

3.1. Good Clinical Practice

This study will be conducted in accordance with Good Clinical Practice (GCP), as defined by the International Conference on Harmonisation (ICH) and in accordance with the ethical principles underlying European Union Directive 2001/20/EC and the United States Code of Federal Regulations, Title 21, Part 50 (21CFR50). The study will be conducted in compliance with the protocol. The protocol and any amendments and the subject informed consent will receive Institutional Review Board/Independent Ethics Committee (IRB/IEC) approval/favorable opinion prior to initiation of the study. All potential serious breaches must be reported to BMS immediately. A serious breach is a breach of the conditions and principles of GCP in connection with the study or the protocol, which is likely to affect, to a significant degree, the safety or physical or mental integrity of the subjects of the study or the scientific value of the study. Personnel involved in conducting this study will be qualified by education, training, and experience to perform their respective tasks. This study will not use the services of study personnel where sanctions have been invoked or where there has been scientific misconduct or fraud (e.g., loss of medical licensure, debarment).

3.2. Institutional Review Board/Independent Ethics Committee

Before study initiation, the investigator must have written and dated approval/favorable opinion from the IRB/IEC for the protocol, consent form, subject recruitment materials (e.g., advertisements), and any other written information to be provided to subjects. The investigator or BMS should also provide the IRB/IEC with a copy of the Investigator Brochure or product labeling information to be provided to subjects and any updates. The investigator or BMS should provide the IRB/IEC with reports, updates and other information (e.g., expedited safety reports, amendments, and administrative letters) according to regulatory requirements or institution procedures.

3.3. Informed Consent

Investigators must ensure that subjects are clearly and fully informed about the purpose, potential risks, and other critical issues regarding clinical studies in which they volunteer to participate.

In situations where consent cannot be given to subjects, their legally acceptable representatives are clearly and fully informed about the purpose, potential risks, and other critical issues regarding

clinical studies in which the subject volunteers to participate. BMS will provide the investigator with an appropriate (i.e., Global or Local) sample informed consent form which will include all elements required by ICH, GCP and applicable regulatory requirements. The sample informed consent form will adhere to the ethical principles that have their origin in the Declaration of Helsinki.

Investigators must:

1. Provide a copy of the consent form and written information about the study. The language must be non-technical and easily understood.
2. Allow time necessary for subject or subject's legally acceptable representative to inquire about the details of the study.
3. Obtain an informed consent signed and personally dated by the subject or the subject's legally acceptable representative and by the person who conducted the informed consent discussion.
4. Obtain the IRB/IEC's written approval/favorable opinion of the written informed consent form and any other information to be provided to the subjects, prior to the beginning of the study, and after any revisions are completed for new information.
5. If informed consent is initially given by a subject's legally acceptable representative or legal guardian, and the subject subsequently becomes capable of making and communicating his or her informed consent during the study, consent must additionally be obtained from the subject.
6. Revise the informed consent whenever important new information becomes available that is relevant to the subject's consent. The investigator, or a person designated by the investigator, should fully inform the subject or the subject's legally acceptable representative or legal guardian, of all pertinent aspects of the study and of any new information relevant to the subject's willingness to continue participation in the study. This communication should be documented.

The confidentiality of records that could identify subjects must be protected, respecting the privacy and confidentiality rules applicable to regulatory requirements, the subjects' signed ICF and, in the US, the subjects' signed HIPAA Authorization.

The consent form must also include a statement that BMS and regulatory authorities have direct access to subject records. Subjects unable to give their written consent (e.g., stroke or subjects with or severe dementia) may only be enrolled in the study with the consent of a legally acceptable representative. The subject must also be informed about the nature of the study to the extent

compatible with his or her understanding, and should this subject become capable, he or she should personally sign and date the consent form as soon as possible. The explicit wish of a subject who is unable to give his or her written consent, but who is capable of forming an opinion and assessing information to refuse participation in or to be withdrawn from, the clinical study at any time should be considered by the investigator. The rights, safety, and well-being of the study subjects are the most important considerations and should prevail over interests of science and society.

4. INVESTIGATIONAL PLAN

4.1. Study Design

This is a Phase I/Ib, non- randomized of concurrent intravenous and intrathecal nivolumab in adult (≥18 years) patients with leptomeningeal disease from melanoma or lung cancer. Patients must have histologically confirmed diagnosis of metastatic melanoma or lung cancer and radiographic and/or CSF cytological evidence of LMD. Patients with prior therapy with anti-PD-1 and/or anti-CTLA-4 will be eligible.

Please see the complete list of inclusion and exclusion criteria in Section 4.2.

4.2. Eligibility Criteria

4.2.1. Inclusion Criteria

1. Patients must have radiographic and/or CSF cytological evidence of LMD.

   For patient with melanoma: Must have a confirmed diagnosis of primary CNS melanoma, melanocytomas or metastatic melanoma (cutaneous, acral-lentiginous, uveal and mucosal in origin), based on histological analysis of metastatic tissue and/or cancer cells, archival tissue permitted.

   For patients with lung cancer: non small cell lung cancer, based on histological analysis of metastatic tissue and/or cancer cells, archival tissue permitted.

2. Patients must have an ECOG PS of ≤ 2.
3. Patients may receive steroids to control symptoms related to CNS involvement, but the dose must be ≤ 4 mg per 24 hours of dexamethasone (or the equivalent). Patient's symptoms should experience stability of neurological symptoms for at least 7 days, or on tapering dose of steroids. Physiologic replacement doses for adrenal insufficiency is allowed on this protocol.
4. Patients who have received radiation to brain and/or spine, including whole brain radiation, stereotactic radiosurgery, or SBRT, are eligible, but must have completed radiation

treatment at least 7 days prior to the start of treatment.

5. Patients who have been treated with an approved targeted therapy (BRAF inhibitor and/or MEK inhibitor) will be allowed to remain on concurrent approved targeted therapy. No other concomitant intrathecal therapy with another agent will be allowed. For patients that have received other systemic therapies, the minimum wash out period is as follows:
   - Patients that received previous IT therapy must have received their last treatment ≥ 7 days prior to the start of treatment
   - Patients who have received systemic chemotherapy must have received their last treatment ≥ 14 days prior to the start of treatment
   - Patients who have received an approved systemic biologic therapy (e.g. anti-PD-1, anti-CTLA4, IL2, interferon) must have received their last treatment ≥ 2 weeks prior to the start of treatment
   - Patients who have received any other investigational agents must have received their last treatment ≥ 14 days prior to the start of treatment
6. For patients with lung cancer:
   - For chemotherapy: patients do not require a washout period, and can continue with chemotherapy during treatment with IT/IV Nivolumab.
   - Patients who have received an approved systemic biologic therapy (e.g. anti-PD-1, anti-CTLA4, IL2, interferon) must have received their last treatment ≥ 2 weeks prior to the start of treatment
   - Patients who have received any other investigational agents must have received their last treatment ≥ 14 days prior to the start of treatment
   - No other concomitant intrathecal therapy with another agent will be allowed.
7. Age ≥ 18 years
8. Capable of giving written informed consent, which includes compliance with the requirements and restrictions listed in the consent form
9. Patients must have organ and marrow function as defined below in Table 1.
10. Absence of contraindication for Ommaya reservoir

Table 1. Eligibility Guidelines of Organ and Marrow Function

System	Laboratory Values
**Hematologic**	
Absolute neutrophil count (ANC)	≥ 1.5 X 10^9/L
Hemoglobin	≥ 9.0 g/dL
Platelets	≥ 75 X 10^9/L
PT/INR and PTT	≤ 1.5 X ULN
**Hepatic**	
Total bilirubin	≤ 1.5 X ULN (isolated bilirubin >1.5 X ULN is acceptable if bilirubin is fractionated and direct bilirubin <35%)
AST and ALT<br><br>Albumin	≤ 2.5 X ULN<br><br>≥2.5 g/dL
**Renal**	
Creatinine OR<br><br>Calculated creatinine clearance OR<br><br>24-hour urine creatinine clearance	≤ 2 X ULN<br><br>≥ 50 mL/min<br><br>≥ 50 mL/min

Women are eligible to participate if:

- Non-childbearing potential defined as pre-menopausal females with a documented tubal ligation or hysterectomy; or postmenopausal defined as 12 months of spontaneous amenorrhea [in questionable cases a blood sample with simultaneous follicle stimulating hormone (FSH) > 40 MlU/mL and estradiol < 40 pg/mL (<140 pmol/L) is confirmatory].
- A Women of childbearing potential (WOCBP) is defined as any female who has experienced menarche and who has not undergone surgical sterilization (hysterectomy or bilateral oophorectomy) and is not postmenopausal. Menopause is defined as 12 months of amenorrhea in a woman over age 45 years in the absence of other biological or physiological causes. In addition, females under the age of 55 years must have a serum follicle stimulating hormone, (FSH) level > 40mIU/mL to confirm menopause.
- *Females treated with hormone replacement therapy, (HRT) are likely to have artificially suppressed FSH levels and may require a washout period in order to obtain a physiologic FSH level. The duration of the washout period is a function of the type of HRT used. The duration of the washout period below are suggested guidelines and the investigators should use their judgment in checking serum FSH levels. If the serum FSH level is >40 mIU/ml at any time during the washout period, the woman can be considered postmenopausal:
- 1 week minimum for vaginal hormonal products (rings, creams, gels)
- 4 week minimum for transdermal products
- 8 week minimum for oral products.
- Other parenteral products may require washout periods as long as 6 months.

- A Women of childbearing potential agrees to use method(s) of contraception. For a teratogenic study drug and/or when there is insufficient information to assess teratogenicity (preclinical studies have not been done), a highly effective method(s) of contraception (failure rate of less than 1% per year) is required.

  The individual methods of contraception and duration should be determined in consultation with the investigator. Women of childbearing potential (WOCBP) must follow instructions for birth control when the half-life of the investigational drug is greater than 24 hours, contraception should be continued for a period of 30 days plus the time required for the investigational drug to undergo five half-lives. The half-life of nivolumab is up to 25 days. WOCBP should use an adequate method to avoid pregnancy for 23 weeks (30 days plus the time required for nivolumab to undergo five half-lives) after the last dose of investigational drug. WOCBP must have a negative serum or urine pregnancy test (minimum sensitivity 25 IU/L or equivalent units of HCG) within 24 hours prior to the start of investigational product.
- Women must not be breastfeeding

Men who are sexually active with WOCBP must use any contraceptive method with a failure rate of less than 1% per year The investigator shall review contraception methods and the time period that contraception must be followed. Men who are sexually active with WOCBP must follow instructions for birth control when the half-life of the investigational drug is greater than 24 hours, contraception should be continued for a period of 90 days plus the time required for the investigational drug to undergo five half-lives. The half-life of nivolumab is up to 25 days. Therefore, men who are sexually active with WOCBP must continue contraception for 31 weeks (90 days plus the time required for nivolumab to undergo five half-lives) after the last dose of investigational drug.

Women who are not of childbearing potential (i.e., who are postmenopausal or surgically sterile and azoospermic men do not require contraception.

**HIGHLY EFFECTIVE METHODS OF CONTRACEPTION**
Male condoms with spermicide
Hormonal methods of contraception including combined oral contraceptive pills, vaginal ring, injectables, implants and intrauterine devices (IUDs) such as Mirena® by WOCBP subject or male subject's WOCBP partner Female partners of male subjects participating in the study may use hormone based contraceptives as one of the acceptable methods of contraception since they will not be receiving study drug:
Nonhormonal IUDs, such as ParaGard®
Tubal ligation
Vasectomy
Complete Abstinence*
*Complete abstinence is defined as complete avoidance of heterosexual intercourse and is an acceptable form of contraception for all study drugs. Subjects who choose complete abstinence are not required to use a second method of contraception, but female subjects must continue to have pregnancy tests. Acceptable alternate methods of highly effective contraception must be discussed in the event that the subject chooses to forego complete abstinence.

LESS EFFECTIVE METHODS OF CONTRACEPTION
Diaphragm with spermicide<br>Cervical cap with spermicide<br>Vaginal sponge<br>Male Condom without spermicide<br>Progestin only pills by WOCBP subject or male subject’s WOCBP partner<br>Female Condom*.<br>* A male and female condom must not be used together

4.2.2. Exclusion Criteria

A subject will not be eligible for inclusion in this study if any of the following criteria apply:

<u>Medical History and concurrent diseases:</u>

1. Patients must not have active autoimmune disease that has required systemic treatment in the past 2 years (i.e., with use of disease modifying agents, corticosteroids or immunosuppressive drugs). Replacement therapy (e.g., thyroxine, insulin, or physiologic corticosteroid replacement therapy for adrenal or pituitary insufficiency, etc.) is not considered a form of systemic treatment
2. Subjects with a condition requiring systemic treatment with either corticosteroids (> 4 mg daily dexamethasone equivalents) or other immunosuppressive medications within 14 days of study drug administration. Inhaled or topical steroids and adrenal replacement doses > 10 mg daily prednisone equivalents are permitted in the absence of active autoimmune disease.
3. Patients who have previously received α-PD-1 and/or anti-CTLA-4 will be eligible, unless they have ongoing >Grade 2 AE side effects of such therapy. Ongoing physiologic replacement doses for adrenal and thyroid insufficiency are allowed on protocol.
4. Currently receiving cancer therapy (chemotherapy, radiation therapy, immunotherapy, or biologic therapy) or investigational anti-cancer drug (concurrent treatment with approved targeted therapies is allowed.)
5. Pregnant or lactating female
6. Subjects with major medical, neurologic or psychiatric condition who are judged as unable to fully comply with study therapy or assessments should not be enrolled.
7. Patients with a history of pneumonitis
8. Evidence of active infection ≤ 7 days prior to initiation of study drug therapy (does not apply to viral infections that are presumed to be associated with the underlying tumor

type required for study entry).

9. Use of non-oncology vaccines containing live virus for prevention of infectious diseases within 12 weeks prior to study drug.

Physical and Laboratory Test Findings

10. Any positive test for hepatitis B virus or hepatitis C virus indicating acute or chronic infection
11. Known history of testing positive for human immunodeficiency virus (HIV) or known acquired immunodeficiency syndrome (AIDS) even if fully immunocompetent on ART—due to the unknown effects of HIV on the immune response to combined nivolumab or the unique toxicity spectrum of these drugs in patients with HIV.

Allergies and Adverse Drug Reaction

12. History of allergy to study drug components.
13. History of severe hypersensitivity reaction to any monoclonal antibody.

Other Exclusion Criteria

14. Prisoners or subjects who are involuntarily incarcerated.
15. Subjects who are compulsorily detained for treatment of either a psychiatric or physical (eg, infectious disease) illness.

4.3. Study Design Schema

This clinical trial will consist of two parts: (1) a dose-escalation portion, and (2) dose-expansion cohort at the recommended Phase Ib dose. Please see Figure 1 and 2 for an overview of the treatment plan for cycles 1 and 2. Each treatment cycle will be 14 days.

Figure 1. Treatment Schedule and Sample Collection- Cycle 1 and Cycle 2:

Cycle	C1															C2													
Day of Cycle	D1	D2	D3	D4	D5	D6	D7	D8	D9	D10	D11	D12	D13	D14		D1	D2	D3	D4	D5	D6	D7	D8	D9	D10	D11	D12	D13	D14
Blood and CSF	x	x						x								x	x						x						
IT nivolumab	x	X														x													
IV nivolumab		X														x													

Figure 2. Treatment Schedule and Sample Collection- Cycle 3 and beyond:

Cycle	C3														C4														C5 and beyond		
Day of Cycle	D1	D2	D3	D4	D5	D6	D7	D8	D9	D10	D11	D12	D13	D14	D1	D2	D3	D4	D5	D6	D7	D8	D9	D10	D11	D12	D13	D14	D1	D2	....D14
Blood and CSF	x														x														x		
IT nivolumab	x														x														x		
IV nivolumab	X														X														X		

4.4. Treatment Assignment

This is a single arm, phase I/Ib trial. No randomization will take place.

4.5. Investigational Product

The investigational product is supplied by BMS. The investigational product should be stored in a secure area according to local regulations. It is the responsibility of the investigator to ensure that investigational product is only dispensed to study subjects. The investigational product must be dispensed only from official study sites by authorized personnel according to local regulations.
In this protocol, investigational product is BMS-936558 (nivolumab).

4.6. Intrathecal Nivolumab

4.6.2. Dose Escalation and Administration of Intrathecal Nivolumab

Previous studies of IT administration of the monoclonal antibodies and trastuzumab most frequently used doses of 10, 25, or 50 mg; rare patients have been safely treated with IT doses of up to 100 or 150 mg. [1-3] The primary objective of the dose escalation portion of the trial is to identify the maximum tolerated dose (MTD) and dose for further study. We have successfully assessed the 3 dose levels of IT nivolumab given every 14 days: 5 mg, 10 mg, and 20 mg (Table 2). We are now planning to assess 50mg of IT Nivolumab (Dose Level 4), as we have not reached the MTD based on safety assessment and analysis. If patients cannot tolerate dose level 4, patients will be enrolled in dose level 3 (Table 2). The intrathecal nivolumab will be prepared in accordance to the IT preparation guidelines for IT Trastuzumab (http://myteams.mdanderson.org/divisions/pharmacy/pharmacy-operations).

In short, the IT dose of nivolumab will be in a maximum volume of 5 ml and in as little as 3 ml qs with preservative free normal saline if needed to complete this volume for administration. The filter used during IT preparation typically is a 0.22 micron filter. The stability of the drug is usually 24 hours refrigerated for IV use and would be 8 hours because of sterility for IT use. The syringe is labeled so the sterile field is broken and the syringe should be placed accordingly during the preparation area for administration.

Either the physician or the midlevel providers will administer the medication. We have treated over 100 patients with intrathecal interleukin-2, which has significant toxicities, and all our inpatient mid-levels are experienced in administration of intrathecal immunotherapy, monitoring of side effects and collection of the CSF.

Table 2: Dose escalation for intrathecal and intravenous nivolumab

Dose level	IT nivolumab q14 days	IV nivolumab q14 days
**-1**	1mg	240mg
**1**	5 mg	240 mg
**2**	10 mg	240 mg
**3**	20 mg	240 mg
**4**	50 mg	240 mg

4.6.1.1. Monitoring of intracranial pressure

We have noted in our experience with intrathecal IL-2 that patients can experience significant toxicities due to increased intracranial pressure (ICP). However, elevated ICP can be typically managed easily by our experienced team of inpatient mid-level providers and nurses. Patients with mild or moderate symptoms due to elevated ICP are typically only treated with supportive medication (acetaminophen, ondansetron, diphenhydramine, metoclopramide, tramadol, meperidine, lorazepam, prochlorperazine and opioid analgesia), while severe symptoms require drainage of CSF via Ommaya.

The same principle of monitoring and management for elevated ICP will be applied for patients receiving treatment with IT nivolumab:

- Intracranial pressure will be measured and documented at bedside each time the Ommaya is accessed.

- In case of mild or moderate symptoms of ICP, supportive medical treatment (acetaminophen, ondansetron, diphenhydramine, metoclopramide, tramadol, meperidine, lorazepam, prochlorperazine and opioid analgesia as needed) will be provided
- In cases of severe symptoms, CSF drainage via Ommaya will be performed, and ICP will be recorded.
- We have also noticed that level of ICP can vary greatly among patients, and therefore, specific cut-off levels for elevated ICP are clinically not useful. Some patients start their first treatment with a level >30, and remain fairly asymptomatic with much higher levels. Some patients only increase their ICP by a few points, however, are much more symptomatic. Therefore, discontinuation of IT nivolumab will not be not based on level of ICP, but on patient's symptoms.
    - If symptoms don't resolve with medical intervention and CSF drainages, subsequent IT dose will be held and medical management and CSF drainage as indicated for symptom management should continue, however, if after holding the IT nivolumab for up to 28 days due to the patient still being too symptomatic, IT nivolumab should be discontinued.
    - Please see 4.11.1 for re-initiation of Intrathecal dosing.

4.6.2. Dose levels

Subjects will receive the intrathecal dose based on the dose level that they were assigned to. There will be no intrasubject dose escalation, and subjects will receive the same IT dose level during all subsequent cycles on an ongoing basis, given tolerability. Intravenous dose is 240mg for all subjects.

We will use the Bayesian mTPI method of Ji et al to find the recommended dose of nivolumab.[20] A maximum of 17 patients will be treated. The dose-finding algorithm is described in detail in the Statistical Considerations section.

We previously had used the following case scenario in the protocol. Given that treatment with IV and IT nivolumab might not produce any DLT, the decision on dose escalation may not be based solely on toxicity, but may also incorporate CSF and/or blood studies, or clinical activity. If no DLT is experienced with the current design, additional dose levels may be considered, and added as a

protocol amendment, based on CSF levels of nivolumab achieved and/or clinical efficacy, in collaboration with BMS.

Based on review of the safety data as well as initial cytokine data, we did not see any significant changes, and no patient experienced IT Nivolumab related toxicities. As we also tested a dose equivalent to 50mg of IT Nivolumab in murine studies, and also did not observe any toxicities, we are now planning to add an additional dose level, as also discussed with the sponsor.

4.6.3. Administration and Timing of IT nivolumab

Subjects will receive IT nivolumab every 14 days. Cycle 1 will consist of IT nivolumab only, but in subsequent cycles the IT nivolumab dose will be followed by an IV dose of nivolumab.

Intrathecal dose will be administered by accessing the Ommaya. Prior to administration of the study drug, CSF will be removed. Minimum CSF amount will be equal to the volume of the study drug, with a maximum of-20ml of CSF during each CSF removal. Dose will be injected over 5 minutes. Needle gauge used will be 25. Bandaid will be placed over the injection site.

As at this time the safety of IT nivolumab is unknown, we will plan that all subjects are admitted to the hospital for all IT treatments during the dose escalation portion of the trial. Starting from cycle 2, IV nivolumab will be administered 4 hours (+/- 1 hour) after the IT Nivolumab dose. Patients who are admitted for observation should receive their IV nivolumab dose while inpatient. For Cycle 1, subjects will remain hospitalized overnight after the IT dosing for safety observation. For Cycle 2 only, subjects will remain hospitalized overnight after the IV infusion for observation with discharge the following day. Beginning with Cycle 3 and all subsequent cycles, subjects will be discharged after the IV Nivolumab infusion after a 30 minute observation period. If the results of the dose escalation support that 24 hour inpatient hospitalization is not required for the management of side effects or patient safety, then the trial may be amended to allow for the administration of IT and IV nivolumab in the outpatient setting for the dose expansion cohort. Dose Limiting Toxicities (DLTs) of IT Nivolumab

DLTs will be recorded. The timeline for assessing DLTs will be for 28 days after the initiation of IT nivolumab (Day 1, Cycle 1). A DLT is defined as any Grade 3 or higher adverse event (AE) during the first 28 days that is at least possibly related to study drug. However, progression of LMD can cause

similar symptoms that can be seen as with intrathecal immunotherapy. Therefore, we will include the toxicities listed in Table 3 ONLY if these symptoms are NOT RESPONSIVE to maximum medical intervention, including CSF removal for elevated intracranial pressure (Table 3) within 96 hours. For patients develop any grade 2 neurotoxicity that persists beyond 14 days and does not improve with medical management, this will be counted as a DLT.

Table 3: Symptoms associated with LMD

Adverse Event	Definition	Grade	Grade Description
Headache	A disorder characterized by a sensation of marked discomfort in various parts of the head, not confined to the area of distribution of any nerve	3 or higher	Severe pain; limiting self care ADL
Cognitive Disturbance	A disorder characterized by a conspicuous change in cognitive function.	3 or higher	Severe cognitive disability; significant impairment of work/school/life performance
Meningismus	A disorder characterized by neck stiffness, headache, and photophobia resulting from irritation of the cerebral meninges.	3 or higher	Severe symptoms; limiting self care ADL
Seizure	A disorder characterized by a sudden, involuntary skeletal muscular contractions of cerebral or brain stem origin.	3 or higher	Multiple seizures despite medical intervention
Somnolence	A disorder characterized by excessive sleepiness and drowsiness	3 or higher	Obtundation or stupor
Vomiting	A disorder marked by forceful discharge of stomach contents	3 or higher	>/= 7 episodes, separated by 5 minutes in 24 hrs, tube feeding, TPN
Fever	A disorder characterized by elevation of the body's temperature above the upper limit of normal.	3 or higher	>40.0 degrees C (>104.0 degrees F) for <=24 hrs

4.6.4. Dose Modifications

Intrapatient dose reductions or dose escalations of intrathecal nivolumab are not permitted.

4.7. Intravenous Nivolumab

Nivolumab, 100 mg/10 mL (10 mg/mL) or 40 mg/mL (10 mg/mL), is to be administered every two weeks at a flat dose of 240 mg as a 30minute IV infusion with a 0.2-micron to 1.2-micron pore size, low-protein binding polyethersulfone membrane in-line filter at the protocol-specified doses. It is not to be administered as an IV push or bolus injection. Nivolumab injection can be infused undiluted (10 mg/mL) or diluted with 0.9% Sodium Chloride Injection, USP or 5% Dextrose Injection, USP to protein concentrations as low as 1 mg/mL. Care must be taken to assure sterility of the prepared solution as the product does not contain any antimicrobial preservative or bacteriostatic agent. At the end of the infusion, flush the line with a sufficient quantity of normal saline. For further details, please refer to the current BMS-936558 (nivolumab) Investigator Brochure.

There are no premedications recommended for nivolumab.

4.7.1. Dose Modifications

Dose reductions or dose escalations of intravenous nivolumab are not permitted.

4.8. Dose Delay Criteria

Because of the potential for clinically meaningful nivolumab-related AEs requiring early recognition and prompt intervention, management algorithms have been developed for suspected AEs of selected categories (Appendix 10.4).

4.9. Intrathecal Dose Delay

If subject develops intolerable central nervous system specific grade 2 toxicities, dose of IT nivolumab can be delayed for up to 28 days. Systemic nivolumab should be held until the IT dosing resumes.
However, IT dosing can continue despite IV dosing being delayed or discontinued (see 4.10. Systemic Dose Delay and 4.11 Criteria to resume treatment).

4.10. Systemic Dose Delay

Nivolumab administration should be delayed for the following:

Any Grade ≥ 2 non-skin, drug-related AE, with the following exceptions:

- Grade 2 drug-related fatigue or laboratory abnormalities do not require a treatment delay
- Any Grade 3 skin, drug-related AE

Any Grade 3 drug-related laboratory abnormality, with the following exceptions for lymphopenia, leukopenia, AST, ALT, total bilirubin, or asymptomatic amylase or lipase:

- Grade 3 lymphopenia or leukopenia does not require dose delay.
- If a subject has a baseline AST, ALT, or total bilirubin that is within normal limits, delay dosing for drug-related Grade ≥ 2 toxicity.
- If a subject has baseline AST, ALT, or total bilirubin within the Grade 1 toxicity range, delay dosing for drug-related Grade ≥ 3 toxicity.

- Any Grade ≥ 3 drug-related amylase or lipase abnormality that is not associated with symptoms or clinical manifestations of pancreatitis does not require dose delay. The Investigator should be consulted for such Grade ≥ 3 amylase or lipase abnormalities.

Any AE, laboratory abnormality, or intercurrent illness which, in the judgment of the investigator, warrants delaying the dose of study medication.

Subjects who require delay of nivolumab should be re-evaluated weekly or more frequently if clinically indicated and resume nivolumab dosing when re-treatment criteria are met.

4.11. Criteria to Resume Treatment

If the criteria to resume treatment are met, the subject should restart treatment at the next scheduled timepoint per protocol. However, if the treatment is delayed past the next scheduled timepoint per protocol, the next scheduled timepoint will be delayed until dosing resumes.

If treatment is delayed or interrupted for > 8weeks, the subject must be permanently discontinued from study therapy, except as specified in discontinuation section.

4.11.1. Intrathecal Dose

Subjects may resume treatment with study drug when the drug-related CNS specific AE(s) resolve to Grade ≤1 or tolerable grade 2 toxicities. In some cases, where drug- related AEs are attributable to the systemic dose, IT dosing can be continued as per study schedule, while IV dose might he held permanently.

4.11.2. Systemic Dose

Subjects may resume treatment with study drug when the drug-related AE(s) attributed to the systemic dose resolve to Grade ≤1 or baseline value, with the following exceptions:

- Subjects may resume treatment in the presence of Grade 2 fatigue
- Subjects who have not experienced a Grade 3 drug-related skin AE may resume treatment in the presence of Grade 2 skin toxicity
- Subjects with baseline Grade 1 AST/ALT or total bilirubin who require dose delays for reasons other than a 2-grade shift in AST/ALT or total bilirubin may resume treatment in the presence

of Grade 2 AST/ALT OR total bilirubin

- Subjects with combined Grade 2 AST/ALT AND total bilirubin values meeting discontinuation parameters should have treatment permanently discontinued
- Drug-related pulmonary toxicity, diarrhea, or colitis, must have resolved to baseline before treatment is resumed. *Subjects with persistent Grade 1 pneumonitis after* completion of a steroid taper over at least 1 month may be eligible for retreatment if investigator allows
- Drug-related endocrinopathies adequately controlled with only physiologic hormone replacement may resume treatment if investigator allows

4.11.3. Discontinuation Criteria

Treatment should be permanently discontinued for the following:

- For intrathecal dosing, any Grade 3 drug-related CNS toxicities that do not respond to medical intervention including medication and CSF drainage. This includes discontinuation of both IT and IV dose.
- Any Grade 2 drug-related uveitis or eye pain or blurred vision that does not respond to topical therapy and does not improve to Grade 1 severity within the re-treatment period OR requires systemic treatment
- For all the toxicities below, IV nivolumab should be permanently discontinued. However, IT nivolumab can be continued as outlined in the study calendar, if the treating physician feels that below drug- related toxicities are due to the IV nivolumab, and not IT nivolumab, and if the patient appears to derive clinical benefit from the IT nivolumab.
- Any Grade 3 non-skin, drug-related adverse event lasting >/=7 days, with the following exceptions for drug-related laboratory abnormalities, uveitis, pneumonitis, bronchospasm, hypersensitivity reactions, and infusion reactions, and endocrinopathies:
- Grade 3 drug-related uveitis, pneumonitis, bronchospasm, hypersensitivity reaction, or infusion reaction of any duration requires discontinuation
- Grade 3 drug-related endocrinopathies adequately controlled with only physiologic hormone replacement do not require discontinuation
- Grade 3 drug-related laboratory abnormalities do not require treatment

discontinuation except those noted below

- Grade 3 drug-related thrombocytopenia >/=7 days or associated with bleeding requires discontinuation
- Any drug-related liver function test (LFT) abnormality that meets the following criteria require discontinuation:
- AST or ALT >/= 8 x ULN
- Total bilirubin >/= 5 x ULN
- Concurrent AST or ALT >/= 3 x ULN and total bilirubin >/= 2 x ULN

- Any Grade 4 drug-related adverse event or laboratory abnormality, except for the following events which do not require discontinuation:
    - Isolated Grade 4 amylase or lipase abnormalities that are not associated with symptoms or clinical manifestations of pancreatitis and decrease to < Grade 4 within 1 week of onset.
    - Isolated Grade 4 electrolyte imbalances/abnormalities that are not associated with clinical sequelae and are corrected with supplementation/appropriate management within 72 hours of their onset
    - Grade 4 lymphopenia or leucopenia
    - Grade 4 drug-related endocrinopathy adverse events, such as adrenal insufficiency, ACTH deficiency, hyper- or hypothyroidism, or glucose intolerance, which resolve or are adequately controlled with physiologic hormone replacement (corticosteroids, thyroid hormones) or glucose-controlling agents, respectively, may not require discontinuation after discussion with and approval from the Investigator [as allowed by protocol]

- Any dosing interruption lasting >/= 8 weeks with the following exceptions:
    - Dosing delays or interruptions to allow for prolonged steroid tapers to manage drug-related adverse events are allowed. Prior to re-initiating treatment in a subject with a dosing interruption lasting >/= 8 weeks, the Investigator must be consulted. Tumor assessments should continue as per protocol even if dosing is interrupted or delayed

      Dosing interruptions or delays lasting >/= 8 weeks that occur for non-drug-related reasons may be allowed if approved by the Investigator. Prior to re-initiating treatment in a subject with a dosing interruption lasting >/= 8 weeks, the Investigator must be consulted. Tumor assessments should continue as per protocol even if dosing is interrupted
- Any adverse event, laboratory abnormality, or intercurrent illness which, in the judgment of the Investigator, presents a substantial clinical risk to the subject with continued nivolumab dosing
- If the treating physician considers ongoing treatment with IT Nivolumab and permanent discontinuation of IV Nivolumab to provide clinical benefit, subjects may continue to be dosed with IT Nivolumab according to the schedule of events. All other requirements of the protocol will remain and be followed with exception of the IV dosing. The investigator must be consulted and agree with continued treatment with IT Nivolumab only and the patient must demonstrate benefit to continue treatment.

4.11.4. Treatment of Nivolumab Related Infusion Reactions

Since nivolumab contains only human immunoglobulin protein sequences, it is unlikely to be immunogenic and induce infusion or hypersensitivity reactions. However, if such a reaction were to occur, it might manifest with fever, chills, rigors, headache, rash, pruritis, arthralgias, hypo- or hypertension, bronchospasm, or other symptoms of allergic-like reactions.

All Grade 3 or 4 infusion reactions should be reported as an SAE if criteria are met. Infusion reactions should be graded according to NCI CTCAE version 4.03 guidelines.

Treatment recommendations are provided below and may be modified based on local treatment standards and guidelines as appropriate:

4.11.4.1. Intrathecal Dose

For Grade 1 symptoms: (Mild reaction; intrathecal administration interruption not indicated; intervention not indicated)

Remain at bedside and monitor subject until recovery from symptoms. The following prophylactic premedications are recommended for future infusions: diphenhydramine 50 mg (or equivalent) and/or acetaminophen 325 to 1000 mg at least 30 minutes before additional nivolumab administrations.

For Grade 2 symptoms: (Moderate reaction requires therapy or infusion interruption but responds promptly to symptomatic treatment [eg, antihistamines, non-steroidal anti-inflammatory drugs, narcotics, IV fluids]; prophylactic medications indicated for 24 hours).

Stop the nivolumab infusion, begin an IV infusion of normal saline, and treat the subject with diphenhydramine 50 mg IV (or equivalent) and/or acetaminophen 325 to 1000 mg; remain at bedside and monitor subject until resolution of symptoms. The following prophylactic premedications are

recommended for future infusions: diphenhydramine 50 mg (or equivalent) and/or acetaminophen 325 to 1000 mg should be administered at least 30 minutes before additional nivolumab administrations.

For Grade 3 or Grade 4 symptoms: (Severe reaction, Grade 3: prolonged [ie, not rapidly responsive to symptomatic medication and/or brief interruption of infusion]; recurrence of symptoms following initial improvement; hospitalization indicated for other clinical sequelae [eg, renal impairment, pulmonary infiltrates]). Grade 4: (life threatening; pressor or ventilatory support indicated).

Immediately discontinue administration of nivolumab. Begin an IV infusion of normal saline, and treat the subject as follows. Recommend diphenhydramine 50 mg IV with methylprednisolone 100 mg IV (or equivalent), as needed. Subject should be monitored until the investigator is comfortable that the symptoms will not recur. Nivolumab will be permanently discontinued. Investigators should follow their institutional guidelines for the treatment of anaphylaxis. Remain at bedside and monitor subject until recovery from symptoms.

4.11.4.2. Systemic Dose

For Grade 1 symptoms: (Mild reaction; infusion interruption not indicated; intervention not indicated)

Remain at bedside and monitor subject until recovery from symptoms. The following prophylactic premedications are recommended for future infusions: diphenhydramine 50 mg (or equivalent) and/or acetaminophen 325 to 1000 mg at least 30 minutes before additional nivolumab administrations.

For Grade 2 symptoms: (Moderate reaction requires therapy or infusion interruption but responds promptly to symptomatic treatment [eg, antihistamines, non-steroidal anti-inflammatory drugs, narcotics, corticosteroids, bronchodilators, IV fluids]; prophylactic medications indicated for 24 hours).

Stop the nivolumab infusion, begin an IV infusion of normal saline, and treat the subject with diphenhydramine 50 mg IV (or equivalent) and/or acetaminophen 325 to 1000 mg; remain at bedside and monitor subject until resolution of symptoms. Corticosteroid or bronchodilator therapy may also be administered as appropriate. If the infusion is interrupted, then restart the infusion at 50% of the original infusion rate when symptoms resolve; if no further complications ensue after 30 minutes,

the rate may be increased to 100% of the original infusion rate. Monitor subject closely. If symptoms recur then no further nivolumab will be administered at that visit. Administer diphenhydramine 50 mg IV, and remain at bedside and monitor the subject until resolution of symptoms. The amount of study drug infused must be recorded on the electronic case report form (eCRF). The following prophylactic premedications are recommended for future infusions: diphenhydramine 50 mg (or equivalent) and/or acetaminophen 325 to 1000 mg should be administered at least 30 minutes before additional nivolumab administrations. If necessary, corticosteroids (recommended dose: up to 25 mg of IV hydrocortisone or equivalent) may be used.

For Grade 3 or Grade 4 symptoms: (Severe reaction, Grade 3: prolonged [ie, not rapidly responsive to symptomatic medication and/or brief interruption of infusion]; recurrence of symptoms following initial improvement; hospitalization indicated for other clinical sequelae [eg, renal impairment, pulmonary infiltrates]). Grade 4: (life threatening; pressor or ventilatory support indicated).

Immediately discontinue infusion of nivolumab. Begin an IV infusion of normal saline, and treat the subject as follows. Recommend bronchodilators, epinephrine 0.2 to 1 mg of a 1:1,000 solution for subcutaneous administration or 0.1 to 0.25 mg of a 1:10,000 solution injected slowly for IV administration, and/or diphenhydramine 50 mg IV with methylprednisolone 100 mg IV (or equivalent), as needed. Subject should be monitored until the investigator is comfortable that the symptoms will not recur. Nivolumab will be permanently discontinued. Investigators should follow their institutional guidelines for the treatment of anaphylaxis. Remain at bedside and monitor subject until recovery from symptoms.

In the case of late-occurring hypersensitivity symptoms (eg, appearance of a localized or generalized pruritis within 1 week after treatment), symptomatic treatment may be given (eg, oral antihistamine, or corticosteroids).

4.11.5. Drug ordering and accountability

BMS is supplying study drug, information in this section may include how to order study drug from BMS or from the Investigator pharmacy. Or this information may be included in a pharmacy manual.

- It is possible that sites may have more than one clinical study on the same drug ongoing at the same time. It is imperative that only drug product designated for this protocol be used for this study

The investigator is responsible for ensuring that the investigational product is stored under the appropriate environmental conditions (temperature, light, and humidity)

If concerns regarding the quality or appearance of the investigational product arise, do not dispense the investigational product, and contact BMS immediately. If commercial investigational product is used, it should be stored in accordance with the appropriate local labeling. All unused drug will be returned and destroyed at the MD Anderson Investigational Pharmacy.

4.11.6. Handling and Dispensing

The investigator should ensure that the study drug is stored in accordance with the environmental conditions (temperature, light, and humidity) as per product information and the Investigator Brochure and per local regulations. It is the responsibility of the investigator to ensure that investigational product is only dispensed to study subjects. The investigational product must be dispensed only from official study sites by authorized personnel according to local regulations. If concerns regarding the quality or appearance of the study drug arise, the study drug should not be dispensed and contact BMS immediately.

Please refer to the current version of the Investigator Brochure for additional information on storage, handling, dispensing, and infusion information for nivolumab.

Infusion-related supplies (e.g., IV bags, in-line filters, 0.9% NaCl solution) will not be supplied by the sponsor and should be purchased locally if permitted by local regulations.

4.11.7. Destruction

Investigator drug destruction is allowed provided the following minimal standards are met:

On-site disposal practices must not expose humans to risks from the drug.

On-site disposal practices and procedures are in agreement with applicable laws and regulations, including any special requirements for controlled or hazardous substances

Written procedures for on-site disposal are available and followed. The procedures must be filed with the Sponsor SOPs and a copy provided to BMS upon request.

Records are maintained that allow for traceability of each container, including the date disposed of, quantity disposed, and identification of the person disposing the containers. The method of disposal, i.e., incinerator, licensed sanitary landfill, or licensed waste disposal vendor must be documented.

Accountability and disposal records are complete, up-to-date, and available for BMS to review throughout the clinical trial period as per the study agreement.

It is the Investigator's responsibility to arrange for disposal of all empty containers, provided that procedures for proper disposal have been established according to applicable federal, state, local, and institutional guidelines and procedures, and provided that appropriate records of disposal are kept.

4.12. Product Descriptions

Nivolumab (Table 4, BMS-936558) vials must be stored at a temperature of 2°C to 8°C and should be protected from light and freezing, and shaking.

Table 4: Product description

**Product Description: (Other names = MDX-1106, ONO-4538, anti-PD-1)**					
**Product Description and Dosage Form**	**Potency**	**Primary Packaging (Volume)/ Label Type**	**Secondary Packaging (Qty) /Label Type**	**Appearance**	**Storage Conditions (per label)**
Nivolumab (BMS-936558-01)* Injection drug product is a sterile, non-pyrogenic, single-use, isotonic aqueous solution formulated at 10 mg/mL	100 mg/Vial (10 mg/mL).	Carton of 5 or 10 vials	10-cc Type 1 flint glass vials stoppered with butyl stoppers and sealed with aluminum seals.	Clear to opalescent, colorless to pale yellow liquid. May contain particles	**BMS-936558-01 Injection** must be stored at 2 to 8 degrees C (36 to 46 degrees F) and protected from light and freezing

*Nivolumab may be labeled as BMS-936558-01 Solution for Injection

If stored in a glass front refrigerator, vials should be stored in the carton.

For additional details on prepared drug storage and use time of nivolumab under room temperature/light and refrigeration, please refer to the BMS-936558 (nivolumab) Investigator Brochure section for “Recommended Storage and Use Conditions”

Care must be taken to assure sterility of the prepared solution as the product does not contain any anti-microbial preservative or bacteriostatic agent. No incompatibilities between nivolumab and polyolefin bags have been observed.

Subjects should be carefully monitored for infusion reactions during nivolumab administration. If an acute infusion reaction is noted, subjects should be managed according to Protocol Section 4.11.4.

Doses of nivolumab may be interrupted, delayed, or discontinued depending on how well the subject tolerates the treatment.

4.12.1. Study Design: Dose expansion cohort

Once the recommended dose for further testing is defined, the trial will continue with an expansion cohort consisting of patients with LMD from melanoma or lung cancer (n=33 patients).

4.12.2. Permanent Discontinuation from Study Treatment and Subject Completion Criteria

4.12.2.1. Subject completion criteria

After subject has stopped receiving nivolumab or if there is an intolerable effect, there will be an End of Treatment Visit. During this visit, a physical exam, blood will be drawn for routine tests and research if tolerated by the patient, CSF will be obtained for research (Table 5) via Ommaya tap.

Every 12 weeks after the subject's last dose, a physical exam will be done. Blood will be drawn for routine tests and if tolerated by the patient, CSF will be obtained via Ommaya tap; an MRI if the brain and spine (if patient able to lay flat and still for the required time), CT scan or PET-CT scan will be done to check the status of LMD as well as extracranial disease.

A subject will be considered to have completed the study after being followed for a minimum of one year after the subject's last dose or the subject is no longer receiving clinical benefit (i.e., has progressive disease per RANO LMD), has died, has shown unacceptable toxicity, or study completion criteria have been met.
. Subjects who are ongoing at the time the study is closed/terminated will be considered to have completed the study.

4.12.2.2. Permanent Discontinuation from Study Treatment

During the protocol defined treatment period study treatment(s) may be permanently discontinued for the following reasons:

- Death
- unacceptable adverse event
- documented disease progression (unless subject meets criteria for treatment beyond progression as defined below)
- deviation(s) from the protocol
- request of the subject or proxy
- investigator's discretion

- subject is lost to follow-up
- study is closed or terminated.

The primary reason the study treatment was permanently discontinued must be documented in the subject's medical records.

If disease recurs prior to the completion of the treatment period, study treatment should be discontinued and follow-up assessments should be conducted every three months. Follow up assessments may be performed in person, by mail or via phone conversation.

If the subject voluntarily discontinues from treatment due to toxicity, 'adverse event' will be recorded as the primary reason for permanently discontinuation in the medical record.

All subjects who permanently discontinue from study treatment will have assessments at the time of discontinuation and during post study treatment follow-up as specified in Time and Events Tables. In addition, all subjects who permanently discontinue study treatment without evidence of disease recurrence will also be followed for disease recurrence according to the protocol schedule until:

- Withdrawal of consent
- Death, or
- Study completion (as defined in Section 4.12.2.1)

Subjects that permanently discontinue from study treatment before the end of the treatment period without evidence of disease recurrence will return for disease assessment visits starting at the next regularly scheduled disease assessment visit (i.e. every 12 weeks for restaging scans). If a subject experiences disease recurrence at any time, subsequent follow up should be conducted every three months in person, by mail or by phone contact.

Follow-up for survival, new anti-cancer therapy (including radiotherapy) and response to new anti-cancer therapy will continue for all subjects including those with disease recurrence for 2 years after the initial dose of IT nivolumab after which all protocol-required assessments and procedures will be discontinued. Follow-up contact to assess survival and new anti-cancer therapy may be made via clinic visit or another form of communication (e.g. phone, email, mail etc.).

4.12.3. Prohibited Medications and Non-drug Therapies

The use of illicit drugs within 28 days or 5 half-lives, whichever is shorter, prior to initiation of IT nivolumab and for the duration of the study will not be allowed.

The following medications or non-drug therapies are also prohibited while on treatment in this study:

- Other anti-cancer therapies (concurrent treatment with approved target therapies is allowed.);
- Other investigational drugs;
- Antiretroviral drugs (Note: Subjects with known HIV are ineligible for study participation);
- Herbal remedies (e.g., St. John's **W**ort)

However, palliative radiation, both to lesions within and outside the CNS, as well as to isolated progressive lesions outside the CNS is allowed on this protocol.

4.12.4. Treatment Beyond Progression

Accumulating evidence indicates a minority of subjects treated with immunotherapy may derive clinical benefit despite initial evidence of PD.

Subjects will be permitted to continue treatment with either intrathecal and systemic nivolumab or only IT nivolumab for their intracranial disease beyond initial RANO LMD criteria defined PD as long as they meet the following criteria determined by the investigator:

- Investigator-assessed clinical benefit and subject is tolerating study drug.
- Tolerance of study drug
- Stable performance status
- Absence of symptoms and signs (including worsening of laboratory values) indicating disease progression.
- Absence of rapid progression of disease.

The assessment of clinical benefit should take into account whether the subject is clinically deteriorating and unlikely to receive further benefit from continued treatment. The disease assessment will continue every 8 weeks (+/- 5 days), and will include MRI imaging, CSF

examination, and neurological exam as part of the ongoing RANO- LMD response criteria. More frequent disease assessments may be performed at the discretion of the investigator.

- Treatment beyond progression will not delay an imminent intervention to prevent serious complications of disease progression.

4.12.5. Isolated systemic disease progression

In the unlikely event of isolated progression of systemic disease while on study, with the LMD remaining stable or improved, the investigator can choose to continue with IT and IV nivolumab, based on evidence that patients with immunotherapy may derive clinical benefit despite initial evidence of PD. This decision will be based on a case by case basis, as long as they meet the following criteria determined by the investigator:

- Investigator-assessed clinical benefit and subject is tolerating study drug.
- Tolerance of study drug
- Stable performance status
- Absence of symptoms and signs (including worsening of laboratory values) indicating disease progression. Absence of rapid progression of disease

5. SCHEDULE OF ASSESSMENTS

5.1 Study Procedure Tables

Table 5: Screening, labs/biopsies, Imaging and Diagnostic tests at baseline and on study

	Baseline/ Screening (28 days)	Cycle 1 (± 3 days)	Cycle 2 (± 3 days)	Cycle 3 (± 3 days)	Cycle 4 (± 3 days)	Cycle 5 (± 3 days)	Every 8 weeks (starting from Cycle 5) (± 3 days)	End of Study Treatment (EOT) (± 3 days)
**General**								
Informed consent	x							
Demographics	x							
Medical history	x							
Concurrent medications	x	x	x	x	x	x	x	
AEs	x	x	x	x	x	x	x	
PE/Vitals/KPS[a]	x	x	x	x	x	x	x	x
**Labs/ Biopsy**								
Pregnancy test[b]	x	x	x	x	x	x	x	
CBC with diff	x	x	x	x	x	x	x	x
Serum chemistry[c]	x	x	x	x	x	x	x	x
TSH, free T4[c]	x	x				x	x	

Liver function profile[d]	x	x	x	x	x	x	x	x
Coagulation	x	x				x	x	
Hepatitis B and C	x							
Research Blood (+/- 2 Days)	x	x	x	x	x	x	x	x
CSF (+/- 2 Days)	x	x	x	x	x	x	x	x
Biopsy[e]	x			x				
**Imaging and Diagnostic tests**								
12-lead EKG	x							
CT CAP or PET (+/- 5 days)	x					x	x	
MRI Brain[f] (+/- 5 days)	x			x		x	x	
MRI Spine[f] (+/- 5 days)	x			x		x	x	
Neurological /cognitive assessment (+/- 5 days)	x			x		x	x	

a. Systolic and diastolic blood pressure, pulse rate, and temperature. Must include full skin examination. Documentation of ECOG or Karnofsky Performance scale is required within 48 hours of each dose of drug administration

b. Serum or urine β-hCG - women of childbearing potential must have a negative pregnancy test within 24 hours prior to the start of investigational product

c. Albumin, bicarbonate, BUN, calcium, chloride, creatinine, glucose, LDH, magnesium, phosphorus, potassium, sodium. Amylase and lipase will be done if clinically indicated. TSH, free T4 will be done at baseline, Cycle 1 and every 8 weeks.

d. Alkaline phosphatase, total bilirubin, total protein, AST, ALT, albumin

e. Optional minimal 5mm punch biopsy or core biopsy of safely accessible lesion.

f. MRI brain and spine at 4 weeks (after cycle 2) and 8 weeks (after Cycle 4). After that, disease assessment will be performed every 8 weeks (+/- 5 days). More frequent disease assessments may be performed at the discretion of the investigator.

Table 6: Nivolumab dosing, ICP monitoring, CSF and blood sampling

	C1 D1	C1 D2	C1 D8	C2 D 1	C2 D2	C2 D8	C3 D1	C4[e] D1	EOT
**CSF[a]**	x	x	x	x	x	x	x	x	x
**Research Blood[b]**	x	x	x	x	x	x	x	x	x
**ICP monitoring[a]**	x	x	x	x	x	x	x	x	x
**Nivolumab**									
IT dose[c]	x			x			x	x	
IV dose[d]				X			x	x	

a. In cycle 1, intracranial pressure will be measured and CSF will be collected before IT nivolumab treatment ~24 hours after treatment; and 7 days after IT treatment. For cycle 2, intracranial pressure will be measured and CSF will be collected before IT nivolumab treatment; at 24 hours after the IT infusion; and 7 days after IT dose 2. In cycle 3, CSF will be collected before IT nivolumab treatment. For patients who continue on combined treatment after cycle 3 and beyond, CSF will be collected prior to IT administration for each cycle. At EOT CSF will be collected and ICP will be monitored.
b. In cycles 1 and 2, blood will be collected at the same timepoints as CSF collections to allow for comparison to CSF analyses. In cycle 1, blood will be collected prior to IT dosing 24 hours, and 7 days after IT dose 1. In cycle 2, blood will be

collected prior to IT administration; 24 hours after IT administration; and 7 days after IT dosing. In cycle 3, blood will be collected before IT nivolumab treatment. For patients who continue on combined treatment, blood will be collected prior to IT administration for each cycle.

c. The first cycle of treatment will consist of IT nivolumab only. Dose escalation schedule for intrathecal nivolumab is shown in Table 2. IT nivolumab is given every 14 days.
d. Intravenous nivolumab dose is always 240mg. It will be administered every 14 days on the same day 4 hours (+/- 1 hour) after IT administration.
e. For patients who continue on combined treatment cycle 4 and beyond, CSF and blood will be collected prior to each cycle.

5.2 Critical Baseline Assessments

Baseline (Screening) assessments will be obtained within 28 days of treatment initiation and will include:

- Complete physical examination, including height (in cm) and weight (in kg)
- Vital signs: blood pressure, temperature, respiratory rate, pulse rate
- Karnofsky Performance Scale or ECOG performance score
- Clinical laboratory tests: hematology, clinical chemistry, coagulation and thyroid function
- Hepatitis B and Hepatitis C screening for acute or chronic infection
- Serum or urine beta-human chorionic gonadotropin (β-HCG) pregnancy test for female subjects of childbearing potential only
- Brain/spine magnetic resonance imaging (MRI) with contrast confirming LMD
- PET/CT or CT of the chest, abdomen and pelvis with contrast. CT of neck if clinically indicated

- CSF baseline sampling, including but not limited to cell count, protein, glucose, cytopathology
- Review of concomitant medications
- Assessment of baseline adverse events
- Acquisition of research blood for immune correlates
- Complete neurological exam
- Neurocognitive assessment, including MoCa and MDASI-BT

Optional:

- Biopsy of accessible tumor for immune correlates

5.3 Safety Evaluations on Therapy

5.3.1 Physical Exams/ECOG/Vital Signs/Concomitant Medications

- A complete physical examination will be performed by a qualified physician or a midlevel provider (physician’s assistant, nurse practitioner). Documentation of Karnofsky Performance Scale status is required within 48 hours of each dose of drug administration. Vital sign measurements will include systolic and diastolic blood pressure, pulse rate, and temperature. Assessment of concomitant medications will be obtained at each clinic visit. The Investigator or physician designee is responsible for providing source documentation and assigning attribution for all AEs.

5.3.2 Adverse Event Assessment

- Assessment of adverse events will be performed at each clinic visit or during stay in the hospital by the research nurse, treating physician or mid-level provider. The Investigator or physician designee is responsible for providing source documentation and assigning attribution for all AEs.

5.3.3 Laboratory assessments

Hematology, clinical chemistry, and additional parameters as per the Study Procedure Tables in section 5.1 to be tested are listed below in Table 7. Laboratory studies must be obtained within 48 hrs prior to drug administration.

Table 7: Required Laboratory Testing

Hematology

Platelet Count	RBC Indices:	Automated WBC Differential:
WBC Count (absolute)	MCV	Neutrophils
Hemoglobin		Lymphocytes
Hematocrit		Monocytes
		Eosinophils
		Basophils

Clinical Chemistry

BUN	Potassium	Sodium	Calcium
Creatinine	Chloride	Magnesium	Phosphorus
Glucose	Total CO_2	Lactate dehydrogenase (LDH)	

Liver function profile

AST (SGOT)	ALT (SGPT)	Total bilirubin
Total protein	Alkaline phosphatase	Albumin

Other tests

β-hCG (urine or serum at screening for females of child bearing potential)
coagulation tests (PT/INR, PTT)
TSH, free T4

5.4 Biospecimen collection

For CSF, blood, and tissue correlative studies specimens will be collected as part of the protocol as specified in the Time and Events Tables (Section 5.1) and stored for later analysis as well as

for future research on an IRB approved protocol. De-linked and de-identified pre-treatment and on treatment tumor tissue specimens will be shared between MD Anderson Cancer Center and Bristol Myers Squibb.

Samples will be stored with the coded identification number or by tissue accession number. All samples and clinical data will be tracked by means of a unique research tracking number that will not be related to any patient identifying information. The link between research tracking numbers and patient identifiers will be kept in a secure application. More than one sample (each sample with a unique research tracking number) may be associated with a single medical record number in the database. Assays will be performed in research laboratories at MD Anderson, MD Anderson Immune Monitoring Core Facility, at Bristol Myers Squibb and as additional outside research laboratories, as appropriate. Results of all assays and other collected data will be maintained in the secure research application.

5.4.1 CSF collection, and intracranial pressure monitoring

CSF collection

CSF will be collected at baseline and EOT (Table 5 and 6). In all subjects, the maximum CSF collected at any given time point is 20 ml, or based on patient's tolerance. CSF will be processed, according to standard operating procedures, and stored at -80°C for subsequent analysis and for future research on an IRB approved protocol.

In cycle 1, CSF will be collected before IT nivolumab treatment; ~ 24 (+/- 1-2 hrs) hours after IT dose;and 7 days after IT treatment. For cycle 2, CSF will be collected before IT nivolumab treatment; at ~24 hours (+/1- 2 hrs) after the IT infusion; and 7 days after IT dose 2. For cycle 3, CSF will be collected before IT nivolumab treatment. For patients who continue on combined treatment after cycle 3, CSF will be collected prior to IT administration for each cycle.

Intracranial pressure monitoring and recording

In cycle 1, intracranial pressure will be measured and recorded before IT nivolumab treatment; ~ 24 (+/- 1-2 hrs) after dose;and 7 days after IT treatment. For cycle 2, intracranial pressure will be measured and recorded before IT nivolumab treatment; at 24 hours (+/1- 2 hrs) after the IT infusion; and 7 days after IT dose 2.

If the results of the dose escalation support that 24 hour inpatient hospitalization is not required for the management of side effects or patient safety, then the trial may be amended to allow for the administration of IT nivolumab in the outpatient setting for the dose expansion cohort. Systemic doses, CSF/blood sampling and ICP monitoring and recording may be done while still in the hospital prior to discharge, or as an outpatient.

5.4.2 Peripheral Blood and Serum Samples

In cycles 1 and 2, blood will be collected at the same time points as CSF collections to allow for comparison to CSF analyses. In cycle 1, blood will be collected prior to IT dosing; ~ 24 hours (=/- 1-2 hours) after IT dose and 7 days after IT dose 1. In cycle 2, blood will be collected prior to IT administration; 24 hours (+/1- 2 hrs) after IT administration; and 7 days after IT dosing. For cycle 3, blood will be collected before IT nivolumab treatment.For patients who continue on combined treatment, blood will be collected prior to IT administration for each cycle. Research blood samples will be collected at the EOT.

The blood will be processed, according to standard operating procedures, for PBMC, cell pellet and plasma. PBMC will be isolated and cryopreserved in 10% DMSO, then stored in liquid nitrogen for subsequent immune and molecular evaluation. Cell pellet will be stored in liquid nitrogen genomic DNA. Plasma will stored at -80°C for future research on an IRB approved protocol.

5.4.3 Tumor Biopsies and Surgical Specimen

If patients are willing and have safely accessible tumors before starting treatment, a tissue biopsy will be performed. These biopsy samples should be excisional, incisional or core needle. Fine needle aspirates or other cytology specimens are insufficient for downstream biomarker analyses. When medically safe and feasible, additional biopsies will be obtained in week 4 after IT nivolumab (+/- 4 days). In addition, local surgery of isolated symptomatic lesions and biopsy prior to re-treatment in the setting of interrupted active treatment is strongly encouraged, if deemed clinically safe. In addition, any patient with accessible tumor at the time of tumor progression, and if safely accessible, will undergo tumor biopsy.

Each set of biopsies will be stored for future correlative analysis.

5.5 Disease Response Assessments

5.5.1 Cytological Response

5.5.1.1. Cytopathology

Cytospins will be prepared in the MDACC Cytology lab to isolate cellular components of CSF. Slides will be assessed visually by an experienced pathologist for the presence of cancer cells to evaluate cytological response. Cytological analysis of CSF will also be assessed every 2 weeks for the first 8 weeks while patients remain on treatment. After that time frame, CSF sampling for cytological response will be performed every 8 weeks. Positive cytology is defined as malignant cells identified in the CSF. Cytological failure will be defined as persistent positive cytology at 8 weeks or the development of positive cytology after two serial negative cytologies.

Available additional slides and CSF may be stored for further research purposes each time the Ommaya is being assessed.

5.5.2. Imaging Assessments

5.5.2.1. CNS imaging

One of the very challenging elements of response assessment in LMD is the neuroimaging evaluation. MRI abnormalities of LM include enhancement of the leptomeninges of the brain or spinal cord identified as enhancement of the cranial nerves and spinal nerve roots, brain surface, cerebellar foliae and within cerebral sulci. [21-23] Enhancement may be nodular, linear or curvilinear as well as focal or diffuse.

Disease assessment will include MRI brain and spine at 4 and 8 weeks (+/- 3 days) after the first dose of IT nivolumab. After that, disease assessment will be performed every 8 weeks (+/- 5 days). More frequent disease assessments may be performed at the discretion of the investigator. Recommend MRI prerequisites and sequences as per the RANO-LMD (per direct communication, manuscript under review) working group are listed below:

Table 8

**MRI prerequisites**
1.5T and 3T MR scanners only
Use of same MRI at baseline and follow-up
MRI to be performed prior to lumbar puncture
**Recommended MRI sequences- Routine MRI brain and spine study sequences to include:**
Brain
Volumetric 3D T1 (MPRAGE or SPGR) post-contrast image with isotropic 1mm voxels to permit reformatting in 3 planes (axial, coronal and sagittal)
Reformatted slice thickness 3mm to obtain good SNR & manageable number of slices for full brain coverage
IV Contrast dose = 0.1mmol/kg of gadolinium-based agent
Spine
Volumetric 3D T1 (MPRAGE or SPGR) post-contrast image in sagittal plane with isotropic 1mm voxels to permit reformatting in 3 planes (axial, coronal and sagittal) with a 2-3mm reformatted slice thickness without gap

Radiographic Assessment will be based on Table 9.

Table 9

MRI findings	Present (1) or absent (0) or non-evaluable (NE)	Dimensions of measurable nodules defined as ≥5 x 10mm (orthogonal diameters)	Change from previous MRI (-3 to +3)
**BRAIN**			
Nodules (subarachnoid or ventricular)			
*Leptomeningeal enhancement			
Cranial nerve enhancement			
Hydrocephalus^			
Parenchymal (brain metastases)^			
**SPINE**			
Nodules (subarachnoid)			
Leptomeningeal enhancement			
Nerve root enhancement			
Parenchymal(intramedullary metastases)^			
Epidural metastasis ^			
**TOTAL SCORE**			

*Leptomeningeal enhancement may include pia, cerebellar folia, ventricular ependyma or cerebral sulci.

^Both hydrocephalus and parenchymal metastases, either brain or spine, are noted as present or absent but not used for LM response determination.

Column 2: scored as 1 (present) or 0 (absent) or non-evaluable (NE). A maximum of 5 radiographic target lesions are selected from baseline imaging to score on follow-up.

Column 3: scores each measurable lesion (at least 5 x 10mm) excluding parenchymal as 1 (present with maximum orthogonal diameters) or 0 (absent).

Column 4: change from baseline or prior image scored as same (0), probable improvement (+1), definite improvement (+2), no evidence of disease (+3) or probable worsening (-1), definite worsening (-2), new site(s) of disease (-3). Measurable nodules defined as ≥5 x 10 mm are scored as same (0), resolved (no evidence of disease, complete response), definitely better (+2; partial response) [decrease by >50% in the summed product of orthogonal diameters], definite worsening (-2; progressive disease) [increase by >25% in the summed product of orthogonal diameters]. A composite score (total score) is calculated and compared to the baseline total score. A 25% worsening in the current score relative to baseline defines radiographic progressive disease. A 50% improvement in the current score defines a radiographic partial response. Resolution of all baseline radiographic abnormalities defines a complete response. All other situations define stable disease.

5.5.3. Neurological Exam and Neurocognitive function test

Clinical response will be considered to be significant improvement of at least 1 neurologic symptom or sign, without deterioration of other symptoms or signs. Any patient with significant neurologic clinical progression (as determined by the treating physician) will be removed from the study, regardless of the status of the other measures.

5.5.3.1. Neurological Exam:

Full neurologic evaluation will be conducted at baseline, at week 4, at week 8, and every 8 weeks thereafter while the patient receives treatment with IT and systemic nivolumab.

The neurologic examination is adapted from RANO Neurological Assessment Group, and is now being used in the RANO LMD evaluation.

Table 10: Neurological examination key points

Domain	Level of Function Score				Key Considerations
	0	1	2	3	
Gait	Normal	Abnormal but walks without assistance	Abnormal and requires assistance (companion, cane, walker, etc.)	Unable to walk	1. Walking is ideally assessed by at least 10 steps.
Strength	Normal	Movement present but decreased against resistance	Movement present but none against resistance	No movement	1. Each limb should be tested separately.<br>2. Recommend assess proximal (above knee or elbow) and distal (below knee or elbow) major muscle groups.<br>3. Score should reflect worst performing area.<br>4. Patients with pre-existing level 3 function in one major muscle group/limb at baseline can be scored based on assessment of other major muscle groups/limb.
Sensation	Normal	Decreased but aware of sensory modality	Unaware of sensory modality	---------	1. Recommend evaluating major body areas separately (face, limbs and trunk).<br>2. Score should reflect worst performing area.<br>3. Sensory modality includes but not limited to light touch, pinprick, temperature and proprioception.<br>4. Patients with pre-existing level 2 function in one major body area at baseline can be scored based on assessment of other major body areas.
Vision	Normal	Partial monocular visual loss	Complete monocular visual loss	Bilateral visual loss	1. Patients who require corrective lenses should be evaluated while wearing corrective lenses<br>2. Each eye should be evaluated and score should reflect worst performing eye.
Eye movements	Normal	Abnormality noted in 1 direction of gaze	Abnormality noted in more than 1 gaze direction, but not all	Unable to move the eye in any gaze direction	1. Test eye movements for each eye individually.<br>2. The score will reflect the worst performing eye (i.e. the highest score.
Facial Strength	Normal	Mild facial weakness (nasolabial fold flattening, asymmetric smile, decreased forehead contraction or partial eye closure)	Severe facial weakness (severe NLF flattening, asymmetric smile with limited or no movement of face, incomplete eye closure, or	Bilateral facial weakness	1Weakness includes nasolabial fold flattening, asymmetric smile and difficulty elevating eyebrow.

			labial incompetence		
Hearing	Normal	Impaired but residual serviceable hearing	Absent unilateral hearing	Bilateral hearing loss	1. Each ear should be evaluated and score should reflect worst performing ear.
Swallowing	Normal	Impaired but not requiring change in diet formulation, not aspirating by bedside testing	Unable to swallow without risk of aspiration by bedside testing	-----------	1. Bedside testing comprised of a swallow test with a small glass of water
Level of Consciousness	Normal	Drowsy (easily arousable & responsive)	Somnolent (difficult to arouse & poorly responsive)	Coma (unarousable & unresponsive)	
Behavior	Normal	Mild/moderate alteration	Severe alteration	---------	1. Alteration includes but is not limited to apathy, disinhibition and confusion.<br>3. Consider subclinical seizures for significant alteration.
Other	Normal	Occasional or mild	Persistent, moderate to severe	------------	

Legend:

Other Neurological findings not otherwise defined in the current examination for example ataxia.

5.5.3.2. Neurocognitive function test

The Montreal Cognitive Assessment (MoCA) and MDASI (MD Anderson Symptom Inventory) will be administered at the same evaluations. These validated tests will allow for monitoring in changes of neurocognitive function and patient-reported neurologic symptoms, respectively, throughout treatment. The MDASI brain tumor module (MDASI-BT) is a site-specific multi-symptom patient-reported outcome measure. Along with the core MDASI's 13 symptom items and 6 interference items, the MDASI-BT also assesses 9 brain-tumor–specific symptom items: weakness on one side of the body, difficulty understanding, difficulty speaking, seizures, difficulty concentrating, vision, change in appearance, change in bowel pattern (diarrhea or constipation), and irritability. Please See Appendix 10.5 for the complete MoCA and MDASI-BT.

5.5.4. Definition of Response

Response of LMD to treatment will be determined by using the RANO LMD criteria. Table 11 below provides the overview for response criteria based on cytology, neurological exam as well as imaging data.

Table 11: RANO-LMD Response criteria

Assessment		Response		Progressive or refractory disease					Stable disease
				Neurological examination defined progression	CSF defined disease progression	Radiologic defined disease progression	Symptoms^		
Neurological exam		Improved		Worse	Stable	Stable	Stable		Stable
CSF									
Cytology<br><br>(all cancers)		Negative		Negative	Positive (lack consensus)	Negative	Negative		Negative or positive (solid tumors only)
CNS<br><br>imaging		Definite Improvement		Stable	Stable	Definite worsening	Stable		Stable or equivocally worsening or improved
Symptom assessment		Improved		Worse or stable	Worse or stable	Worse or stable	Worse		Stable

Legend:

CSF cytology negative	Defined as either true negative or atypical
CSF cytology positive	Defined as true positive or suspicious
Stable	Defined as stable or indeterminate
Symptoms^	Stable; no change (-1 to +1 in symptom inventory
	Worse; -2 to -3 in symptom inventory
	Improved; +2 to +3 in symptom inventory

5.5.4.1. Monitoring of extracranial disease

CT CAP or PET/CT will be performed at the same time points as the MRI brain and spine and will used to monitor the extracranial disease status. Extracranial response will be evaluated using the RESIST 1.1 Criteria (See Appendix 10.1).

5.5.5. Translational Research/Biomarker Analysis

For blood and tissue correlative studies, specimens will be collected as part of the protocol and stored for later analysis and/or future use on approved IRB protocols. All human specimens will be stored on site unless specifically described. Analyses may be conducted at MD Anderson or additional outside research lab facility as appropriate.

5.5.5.1. Pharmacokinetics/ Pharmacodynamics

Levels of nivolumab will be measured in both blood and CSF biospecimens collected at different time points. Once the appropriate assay for PK/PD monitoring of nivolumab is identified, stored samples will be analyzed by BMS.

5.5.5.2. Correlative analysis

Peripheral blood will be collected at the same time points as CSF. For blood and CSF, we plan to perform but not limited to the following analysis: immunohistochemistry, cytokine and nanostring analysis.

5.5.5.3. Immune monitoring

For immune monitoring of blood and CSF, we plan to perform but not limited to the following analysis: flow cytometry as well as TCR seq analysis.

6 SAFETY AND REPORTING

The investigator and site staff will be responsible for detecting, documenting and reporting events that meet the definition of an adverse event (AE) or serious adverse event (SAE). Subjects will be monitored continuously for AEs throughout the study. Subjects must be instructed to notify their physician immediately for any and all toxicities.

6.1 Serious Adverse Event Reporting (SAE) Language

An adverse event or suspected adverse reaction is considered "serious" if, in the view of either the investigator or the sponsor, it results in any of the following outcomes:

- Death
- A life-threatening adverse drug experience – any adverse experience that places the patient, in the view of the initial reporter, at immediate risk of death from the adverse experience as it occurred. It does not include an adverse experience that, had it occurred in a more severe form, might have caused death.
- Inpatient hospitalization or prolongation of existing hospitalization
- A persistent or significant incapacity or substantial disruption of the ability to conduct normal life functions.
- A congenital anomaly/birth defect.
- Important medical events that may not result in death, be life-threatening, or require hospitalization may be considered a serious adverse drug experience when, based upon appropriate medical judgment, they may jeopardize the patient or subject and may require medical or surgical intervention to prevent one of the outcomes listed in this definition. Examples of such medical events include allergic bronchospasm requiring intensive treatment in an emergency room or at home, blood dyscrasias or convulsions that do not result in inpatient hospitalization, or the development of drug dependency or drug abuse (21 CFR 312.32).

- Important medical events as defined above, may also be considered serious adverse events. Any important medical event can and should be reported as an SAE if deemed appropriate by the Principal Investigator or the IND Sponsor, IND Office.
- All events occurring during the conduct of a protocol and meeting the definition of a SAE must be reported to the IRB in accordance with the timeframes and procedures outlined in "The University of Texas M. D. Anderson Cancer Center Institutional Review Board Policy for Investigators on Reporting Unanticipated Adverse Events for Drugs and Devices". Unless stated otherwise in the protocol, all SAEs, expected or unexpected, must be reported to the IND Office, regardless of attribution (within 5 working days of knowledge of the event).
- All life-threatening or fatal events, that are unexpected, and related to the study drug, must have a written report submitted within 24 hours (next working day) of knowledge of the event to the Safety Project Manager in the IND Office.
- Unless otherwise noted, the electronic SAE application (eSAE) will be utilized for safety reporting to the IND Office and MDACC IRB.
- Serious adverse events will be captured from the time of the first protocol-specific intervention, until 100 days after the last dose of drug, unless the participant withdraws consent. Serious adverse events must be followed until clinical recovery is complete and laboratory tests have returned to baseline, progression of the event has stabilized, or there has been acceptable resolution of the event.
- Additionally, any serious adverse events that occur after the 100 day time period that are related to the study treatment must be reported to the IND Office. This may include the development of a secondary malignancy.

6.2 Reporting to FDA:

- Serious adverse events will be forwarded to FDA by the IND Sponsor (Safety Project Manager IND Office) according to 21 CFR 312.32.

It is the responsibility of the PI and the research team to ensure serious adverse events are reported according to the Code of Federal Regulations, Good Clinical Practices, the protocol guidelines, the sponsor's guidelines, and Institutional Review Board policy.

6.3 Investigator Communication with Supporting Company BMS:

6.3.1 SAE Reporting

- All Serious Adverse Events (SAEs) that occur following the subject's written consent to participate in the study through end of treatment must be reported to BMS Worldwide Safety. In addition, any study drug related SAEs occurring up to 100 days of discontinuation of dosing should also be reported to BMS Worldwide Safety.
- If the BMS safety address is not included in the protocol document (e.g. multicenter studies where events are reported centrally), the procedure for safety reporting must be reviewed/approved by the BMS Protocol Manager. Procedures for such reporting must be reviewed and approved by BMS prior to study activation.
- MD Anderson SAE form will be used to report SAEs.
- Following the subject's written consent to participate in the study, all SAEs, whether related or not related to study drug, are collected, including those thought to be associated with protocol-specified procedures. The investigator should report any SAE occurring after these time periods that is believed to be related to study drug or protocol-specified procedure.The Sponsor Investigator will reconcile the clinical database SAE cases transmitted to BMS Global Pharmacovigilance (Worldwide.Safety@bms.com).

- The Investigator will request from BMS GPV&E, aepbusinessprocess@bms.com the SAE reconciliation report and include the BMS protocol number every 3 months and prior to data base lock or final data summary

- GPV&E will send the investigator the report to verify and confirm all SAEs have been transmitted to BMS GPV&E.

- The data elements listed on the GPV&E reconciliation report will be used for case identification purposes. If the Investigator determines a case was not transmitted to BMS

GPV&E, the case should be sent immediately to BMS (Worldwide.Safety@bms.com).

- In accordance with local regulations, BMS will notify investigators of all reported SAEs that are suspected (related to the investigational product) and unexpected (i.e., not previously described in the IB). In the European Union (EU), an event meeting these criteria is termed a Suspected, Unexpected Serious Adverse Reaction (SUSAR).

Investigator notification of these events will be in the form of an expedited safety report (ESR).

- Other important findings which may be reported by the as an ESR include: increased frequency of a clinically significant expected SAE, an SAE considered associated with study procedures that could modify the conduct of the study, lack of efficacy that poses significant hazard to study subjects, clinically significant safety finding from a nonclinical (e.g., animal) study, important safety recommendations from a study data monitoring committee, or sponsor decision to end or temporarily halt a clinical study for safety reasons.
- Upon receiving an ESR from BMS, the investigator must review and retain the ESR with the IB. Where required by local regulations or when there is a central IRB/IEC for the study, the sponsor will submit the ESR to the appropriate IRB/IEC. The investigator and IRB/IEC will determine if the informed consent requires revision. The investigator should also comply with the IRB/IEC procedures for reporting any other safety information.
- In addition, suspected serious adverse reactions (whether expected or unexpected) shall be reported by BMS to the relevant competent health authorities in all concerned countries according to local regulations (either as expedited and/or in aggregate reports).

6.3.2 Serious Adverse Event Collection and Reporting

Following the subject’s written consent to participate in the study, all SAEs, whether related or not related to study drug, must be collected, including those thought to be associated with protocol-specified procedures. All SAEs must be collected that occur within 100 days of discontinuation of dosing SAEs related to study drug will be reported to BMS.

All SAEs must be collected that occur during the screening period. If applicable, SAEs must be collected that relate to any protocol-specified procedure (e.g., a follow-up skin biopsy). The

investigator should report any SAE that occurs after these time periods that is believed to be related to study drug or protocol-specified procedure.

SAEs, whether related or not related to study drug, and pregnancies must be reported to BMS within 24 hours. SAEs must be recorded on BMS or an approved form; pregnancies on a Pregnancy Surveillance Form.

SAE Email Address: Worldwide.Safety@BMS.com

SAE Facsimile Number: 609-818-3804

If only limited information is initially available, follow-up reports are required. (Note: Follow-up SAE reports should include the same investigator term(s) initially reported.)

If an ongoing SAE changes in its intensity or relationship to study drug or if new information becomes available, a follow-up SAE report should be sent within 24 hours to the BMS (or designee) using the same procedure used for transmitting the initial SAE report.

All SAEs should be followed to resolution or stabilization.

For studies conducted under an Investigator IND in the US include the following:

For studies conducted under an Investigator IND in the US, any event that is both serious and unexpected must be reported to the Food and Drug Administration (FDA) as soon as possible and no later than 7 days (for a death or life-threatening event) or 15 days (for all other SAEs) after the investigator's or institution's initial receipt of the information. BMS will be provided with a simultaneous copy of all adverse events filed with the FDA.

SAEs should be reported on MedWatch Form 3500A, which can be accessed at:

http://www.accessdata.fda.gov/scripts/medwatch/.

MedWatch SAE forms should be sent to the FDA at:

MEDWATCH

5600 Fishers Lane

Rockville, MD 20852-9787

Fax: 1-800-FDA-0178 (1-800-332-0178)

http://www.accessdata.fda.gov/scripts/medwatch/

All SAEs should simultaneously be faxed or e-mailed to BMS at:

Global Pharmacovigilance & Epidemiology

Bristol-Myers Squibb Company

Fax Number: 609-818-3804

Email: Worldwide.safety@bms.com

- An SAE report should be completed for any event where doubt exists regarding its seriousness.
- For studies with long-term follow-up periods in which safety data are being reported, include the timing of SAE collection in the protocol.
- If the investigator believes that an SAE is not related to study drug, but is potentially related to the conditions of the study (such as withdrawal of previous therapy or a complication of a study procedure), the relationship should be specified in the narrative section of the SAE Report Form.
- If only limited information is initially available, follow-up reports are required. (Note: Follow-up SAE reports should include the same investigator term(s) initially reported.)
- If an ongoing SAE changes in its intensity or relationship to study drug or if new information becomes available, a follow-up SAE report should be sent within 24 hours to BMS using the same procedure used for transmitting the initial SAE report. All SAEs should be followed to resolution or stabilization. All SAEs should be followed to resolution or stabilization.

6.3.3 Non-serious Adverse Event Collection and Reporting

The collection of non-serious AE information should begin at initiation of study drug. All non-serious adverse events (not only those deemed to be treatment-related) should be collected continuously during the treatment period. Treatment related adverse events should be collected for a minimum of 100 days following the last dose of study treatment.

Non-serious AEs should be followed to resolution or stabilization, or reported as SAEs if they become serious. Follow-up is also required for non-serious AEs that cause interruption or discontinuation of study drug and for those present at the end of study treatment as appropriate. These non-serious AEs will be retained in the Prometheus Database.

6.3.4 Laboratory Test Abnormalities

All laboratory test results captured as part of the study should be recorded following institutional procedures. Test results that constitute SAEs should be documented and reported as such.

The following laboratory abnormalities should be documented and reported appropriately:

- any laboratory test result that is clinically significant or meets the definition of an SAE
- any laboratory abnormality that required the subject to have study drug discontinued or interrupted
- any laboratory abnormality that required the subject to receive specific corrective therapy.

6.3.5 Pregnancy

If, following initiation of the investigational product, it is subsequently discovered that a study subject is pregnant or may have been pregnant at the time of investigational product exposure, including during at least 6 half-lives after product administration, the investigational product will be permanently discontinued in an appropriate manner (e.g., dose tapering if necessary for subject safety).

The investigator must immediately notify Worldwide.safety@bms.comof this event via the Pregnancy Surveillance Form in accordance with SAE reporting procedures.

Follow-up information regarding the course of the pregnancy, including perinatal and neonatal outcome and, where applicable, offspring information must be reported on the Pregnancy Surveillance Form [provided upon request from BMS]

Any pregnancy that occurs in a female partner of a male study participant should be reported to BMS. Information on this pregnancy will be collected on the Pregnancy Surveillance Form.

6.3.6 Overdose

An overdose is defined as the accidental or intentional administration of any dose of a product that is considered both excessive and medically important. All occurrences of overdose must be reported as an SAE.

6.3.7 Other Safety Considerations

Any significant worsening noted during interim or final physical examinations, electrocardiograms, x-rays, and any other potential safety assessments, whether or not these procedures are required by the protocol, should also be recorded as a nonserious or serious AE, as appropriate, and reported accordingly.

7 STATISTICAL CONSIDERATIONS

This is a Phase Ib study of IT and IV nivolumab in patients with metastatic melanoma. There will be two parts to the study: a dose escalation part followed by a dose expansion part. We expect to enroll no more than 17 patients in the dose escalation part of the trial and 33 patients in the dose-expansion part. The primary objective of the dose escalation part of the trial will be the determination of the recommended dose of combined IT and IV nivolumab for further testing. The Bayesian mTPI method will be used to find the recommended dose. For the expansion cohort, the primary objective will be to estimate overall survival with combined IT and IV nivolumab in patients with LMD.

7.1 Dose Escalation Part

The following was the original design of the dose-escalation part of the study. We will use the Bayesian mTPI method of Ji et al to find the MTD dose of nivolumab.[20] Dose- limiting toxicity will be defined as the development of grade 3 or higher CNS toxicity or systemic toxicity. The targeted maximum DLT rate is 30%, and we will enroll patients in cohorts of size 2. We assume a prior distribution of Beta (1, 1) for the DLT rate at each dose level, and we also assume an equivalence interval around the DLT rate of 5% in either direction; i.e., (25%, 35%).
A maximum of 17 patients will be treated. The figure (Fig 4) below provides the rules for escalation and de-escalation once each cohort of 2 patients has been assessed.

Given the nature of this clinical trial, the number of patients simultaneously exposed to a potential safety risk with IT administration of nivolumab should be limited to minimize this risk. Therefore, we will treat patients not simultaneously at each dose level, but stagger each subsequent patient with a minimum of 48 hours at each dose level.
Figure 4 provides the dose escalation algorithm for the study. Figure 5 summarizes the operating characteristics of the design for this trial for 6 scenarios defined by different toxicity rates for the three doses. These operating characteristics are based on 2000 simulations of the trial.

Given that treatment with IV and IT nivolumab might not produce any DLT, the decision on dose escalation may not be based solely on toxicity, but may also incorporate CSF and/or blood studies, or clinical activity, with an overall survival of > 6 months in >/= 50% of patients. If no DLT is experienced with the current design, additional dose levels may be considered, and added as a protocol amendment, based on CSF levels of nivolumab achieved and/or clinical efficacy, in collaboration with BMS. These additional dose levels would only affect the intrathecal nivolumab dose, with the systemic IV dose of nivolumab remaining at a flat dose of 240 mg every 2 weeks.

A Toxicity Summary will be submitted to the IND Office Medical Monitor after the first 2 evaluable patients, and every 2 evaluable patients thereafter (prior to dose escalation or dose expansion).

Fig. 3

Figure 4 . Dose-Escalation Rules

Number of dose limiting toxicities (DLT's)	2	4	6	8	10	12	14	16	18
0	E	E	E	E	E	E	E	E	E
1	S	S	E	E	E	E	E	E	E
2	DU	S	S	S	S	E	E	E	E
3		DU	S	S	S	S	S	E	E
4		DU	DU	D	S	S	S	S	S
5			DU	DU	D	S	S	S	S
6			DU	DU	DU	D	S	S	S
7				DU	DU	DU	D	S	S
8				DU	DU	DU	DU	DU	S
9					DU	DU	DU	DU	DU
10					DU	DU	DU	DU	DU
11						DU	DU	DU	DU
12						DU	DU	DU	DU
13							DU	DU	DU
14							DU	DU	DU
15								DU	DU
16								DU	DU
17									DU
18									DU

E = Escalate to the next higher dose
S = Stay at the current dose
D = De-escalate to the next lower dose
U = The current dose is unacceptably toxic
Targeted Maximum Toxicity = 30%
Sample Size = 18
Epsilon1 = 0.05
Epsilon2 = 0.05

Figure 5 Operating Characteristics of mTPI Phase I Study Design

Dose Level	1	2	3	No Dose
**Scenario 1**				
True Toxicity Rate	0.025	0.05	0.10	
Selection Probability	0.5%	1.6%	97.9%	0.1%
Avg. # of Patients Treated	2.3	2.6	13.1	
Overall Toxicity Rate	0.082			
**Scenario 2**				
True Toxicity Rate	0.05	0.15	0.25	
Selection Probability	3.8%	20.3%	75.5%	0.5%
Avg. # of Patients Treated	3.1	5.0	9.9	
Overall Toxicity Rate	0.190			
**Scenario 3**				
True Toxicity Rate	0.05	0.20	0.35	
Selection Probability	6.5%	39.7%	53.3%	0.7%
Avg. # of Patients Treated	3.3	6.7	7.9	
Overall Toxicity Rate	0.241			
**Scenario 4**				
True Toxicity Rate	0.25	0.35	0.45	
Selection Probability	38.4%	33.4%	16.8%	11.5%
Avg. # of Patients Treated	8.0	5.5	2.9	
Overall Toxicity Rate	0.320			
**Scenario 5**				
True Toxicity Rate	0.35	0.45	0.55	
Selection Probability	49.4%	19.8%	4.1%	26.8%
Avg. # of Patients Treated	9.8	3.9	1.1	
Overall Toxicity Rate	0.391			
**Scenario 6**				
True Toxicity Rate	0.10	0.30	0.50	
Selection Probability	19.3%	58.9%	19.9%	2.0%
Avg. # of Patients Treated	5.2	8.4	4.2	
Overall Toxicity Rate	0.287			

Figure 4

		2	4	6	8	10	12	14	16	18
Number of dose limiting toxicities (DLT's)	0	E	E	E	E	E	E	E	E	E
	1	S	S	E	E	E	E	E	E	E
	2	DU	S	S	S	S	E	E	E	E
	3		DU	S	S	S	S	S	E	E
	4		DU	DU	D	S	S	S	S	S
	5			DU	DU	D	S	S	S	S
	6			DU	DU	DU	D	S	S	S
	7				DU	DU	DU	D	S	S
	8				DU	DU	DU	DU	DU	S
	9					DU	DU	DU	DU	DU
	10					DU	DU	DU	DU	DU
	11						DU	DU	DU	DU
	12						DU	DU	DU	DU
	13							DU	DU	DU
	14							DU	DU	DU
	15								DU	DU
	16								DU	DU
	17									DU
	18									DU

Figure 4: Dose-Escalation Rules

E = Escalate to the next higher dose
S = Stay at the current dose
D = De-escalate to the next lower dose
U = The current dose is unacceptably toxic
Targeted Maximum Dose-Limiting Toxicity = 30%
Sample Size = 18
Epsilon1 = 0.025
Epsilon2 = 0.025

The MTD is defined as the highest dose for which the posterior probability of DLT is closest to 30%. If during the course of the trial, there is little or no DLT experienced, and patients are being treated at dose level 3, the study chair, in consultation with the study biostatistician, may decide to treat fewer than 18 total patients in the dose escalation portion of the study, provided that at least 6 total patients have been treated at dose level 3. The study chair may also, in consultation with the study biostatistician and BMS, add one or more higher dose levels to be studied. If this occurs, the protocol will be amended as necessary to allow for additional patients and dose levels.

Figure 5 below summarizes the operating characteristics of the design for this trial for 6 scenarios defined by different DLT rates for the three doses. These operating characteristics are based on 2000 simulations of the trial.

March 2020 Update: the original dose-escalation part of the trial ended with 11 total patients treated, 6 at the highest dose level, 20 mg. The dose-expansion part began, and 5 patients have been treated to date with no DLT, with 2 additional patients planned to be enrolled shortly. As noted above, after consultation with the sponsor, the study team has decided to add an additional level, dose level 4 at 50 mg. The trial will revert to the mTPI design to test patients at this dose level. If the decision from the mTPI algorithm at any point is to de-escalate, the dose expansion part will restart using dose level 3 to treat patients. Otherwise, the dose-escalation part will continue to treat patients at dose level 4. If there is little or no DLT experienced at this dose, the dose expansion part will start anew at this dose, providing that a minimum of 6 patients have been treated. (See below for additional details.) The operating characteristics will not be updated based upon the addition of dose level 4.

Fig. 5

**Dose Level**	**1**	**2**	**3**	**No Dose**
**Scenario 1**				
True DLT Rate	0.025	0.05	0.10	
Selection Probability	0.4%	2.1%	97.5%	0.1%
Avg. # of Patients Treated	2.3	2.6	13.1	
Overall DLT Rate	0.085			
**Scenario 2**				
True DLT Rate	0.05	0.15	0.25	
Selection Probability	4.0%	20.4%	75.5%	0.3%
Avg. # of Patients Treated	3.0	5.0	10.0	
Overall DLT Rate	0.190			
**Scenario 3**				
True DLT Rate	0.05	0.20	0.35	
Selection Probability	7.0%	38.9%	53.7%	0.5%
Avg. # of Patients Treated	3.4	6.7	7.9	
Overall DLT Rate	0.241			
**Scenario 4**				
True DLT Rate	0.25	0.35	0.45	
Selection Probability	38.5%	35.7%	15.2%	10.8%
Avg. # of Patients Treated	8.1	5.6	2.8	
Overall DLT Rate	0.322			
**Scenario 5**				
True DLT Rate	0.35	0.45	**0.55**	
Selection Probability	49.7%	20.1%	**4.6%**	**24.8%**

Avg. # of Patients Treated	9.5	4.1	**1.3**	
Overall DLT Rate	0.384			
**Scenario 6**				
True DLT Rate	0.10	0.30	**0.50**	
Selection Probability	19.7%	58.8%	**20.3%**	**1.3%**
Avg. # of Patients Treated	5.1	8.6	**4.1**	
Overall DLT Rate	0.290			

Fig 5: Operating Characteristics of mTPI Phase I Study Design

7.2 Dose Expansion

Note: the following three paragraphs were the original plan for the dose-expansion cohort.

Once the recommended dose for further testing is defined, the trial will continue with an expansion cohort consisting of patients with LMD from melanoma and lung cancer.

The primary objective of the dose expansion part of the trial will be to estimate OS in patients treated with IT and IV nivolumab. Patients with LMD from the dose-escalation part of the trial treated at the recommended dose will be included with the additional 12 patients. Minimum expected follow up for overall survival at the time of final analysis is 12 months.

Continuous monitoring of toxicity will be assessed for the 12 patients in the dose-expansion phase of the trial beginning with the first three patients. Assuming a prior beta distribution of (0.6, 1.4), corresponding to a DLT rate of 30%, treatment will terminate if the Pr ($\delta_t > 0.30$ | data) > 0.90, where δ_t is the DLT rate attributable to the treatment.

March 2020 update: A total of 5 patients have been treated at dose level 3 (20 mg) in this dose-expansion part with little or no toxicity, with 2 patients scheduled to enroll shortly. As noted above, we are adding an additional dose level to be tested in the dose-escalation phase. If this dose is found to be safe with little or no DLT in 6 patients, we now plan to treat an additional 26 patients in the dose-expansion part. A new monitoring rule for this part is found below. Note that after the addition of dose level 4, if the decision is made to treat dose expansion patients at this level, the rule will start with the first patient. However, if dose level 4 proves too toxic and the decision is made to restart patients at dose level 3 in the dose-expansion part, the monitoring rule below will continue with the 8th patient, including the 7 that will have already enrolled into this part.

Assuming that the dose-expansion phase starts anew at the 50 mg dose, the new toxicity monitoring rule is as follows: patients will be monitored in cohorts of size 3. Assuming a prior beta distribution of (0.6, 1.4), corresponding to a DLT rate of 30%, enrollment into the dose-expansion part will terminate if Pr ($\delta_t > 0.30$ | data) > 0.95, where δ_t is the DLT rate attributable to the treatment. The boundaries corresponding to this rule are presented in Table 12, and the operating characteristics for this rule are presented in Table 13. The method used to produce the decision rule and operating characteristics was designed by Thall, Simon, and Estey, and the software used was MultcLean Desktop version 2.1.0.

Table 1**2**. Stopping Criteria (Assuming Dose Expansion Restarts Using 50 mg Dose)

**Total Patients Enrolled**	**Stopping Condition** (# of patients experiencing DLTs)
3	3
6	5-6
9	6-9
12	7-12
15	8-15
18	9-18
21	11-21
24	12-24
26	Patient enrollment ends

Table **13**. Operating Characteristics (Assuming Dose Expansion Restarts Using 50 mg Dose)

True % of DLTs	Pr (stopping early)	Average # of patients treated
0.10	0.001	26.0
0.20	0.016	25.7
0.30	0.107	24.3
0.40	0.363	21.6
0.50	0.707	15.2
0.60	0.929	10.1

However, if the 50 mg dose proves too toxic, then the additional 26 patients will be treated at the 20 mg dose, which will restart with the 8th patient (the first patient in the new dose-expansion phase). The toxicity monitoring rule to be used will be is as follows: patients will be monitored in cohorts of size 3. Assuming a prior beta distribution of (0.6, 1.4), corresponding to a DLT rate of 30%, enrollment into the dose-expansion part will terminate if Pr ($\delta_t > 0.30$ | data) > 0.95, where δ_t is the DLT rate attributable to the treatment. The boundaries corresponding to this rule are presented in Table 14, and the operating characteristics for this rule are presented in Table 15. The method used to produce the decision rule and operating characteristics was designed by Thall, Simon, and Estey, and the software used was MultcLean Desktop version 2.1.0.

Table 14. Stopping Criteria Assuming Dose Expansion Continues to Patient 33 Using 20 mg dose

**Total Patients Enrolled**	**Stopping Condition (# of patients experiencing DLTs)**
3	3
6	5-6
9	6-9
12	7-12
15	8-15
18	9-18
21	11-21
24	12-24
27	13-27
30	14-30
33	Patient enrollment ends

Table **15**. Operating Characteristics Assuming Dose Expansion Continues to Patient 33 Using 20 mg dose

True % of DLTs	Pr (stopping early)	Average # of patients treated
0.10	0.001	33.0
0.20	0.016	32.6
0.30	0.119	30.6
0.40	0.425	25.2
0.50	0.793	17.5
0.60	0.969	11.4

Secondary objectives that will be assessed in all LMD patients treated at the RECOMMENDED DOSE include:

1. Overall Survival: The Kaplan-Meier method will be used to estimate the distribution of OS from the start of study treatment, and Cox proportional hazard regression will be used to assess the relationship between OS and various covariates of interest, including but not limited to patient demographics, tumor characteristics, disease characteristics, and the expression of biomarkers.
2. Immunological Effects of Nivolumab

The investigator is responsible for submitting efficacy/toxicity summary to the IND Office as below.

Escalation phase:

This should be submitted after the first 2 evaluable patients complete 28 days, and every 2 evaluable patients thereafter. Approval must be obtained prior to dose escalation or dose expansion.

Expansion phase :

This should be submitted after the first 3 evaluable patients complete 28 days, and every 3 evaluable patients thereafter. Please update the toxicity and survival information of the previously submitted patients during every submission.

A copy of the cohort summary should be placed in the Investigator's Regulatory Binder under "sponsor correspondence".

7.3 Patient Characteristics

Demographic and baseline characteristics of the ITT population will be summarized for all patients. Categorical measures will be summarized using frequencies and percentages while continuous variables will be summarized using mean, standard deviation, median, minimum, and maximum.

Clinical responses will be assessed at 8 weeks, and at 16 and 24 weeks for patients who continue treatment, and they will be characterized as follows:

- *Complete response:* disappearance of the symptoms associated with LMD, cessation of steroid use, and disappearance of radiologic or CSF findings that led to diagnosis of LMD
- *Partial Response:* Improvement in all features noted above but without complete disappearance
- *Stable Disease:* No change or partial improvement in some of the variables
- *Progression:* Worsening of at least one of the variables noted above

7.4 Exploratory Efficacy Measures

Various molecular markers with potential predictive value for the treatment of melanoma may be assessed.

In addition, subsequent lines of therapy will be reported and evaluated using descriptive analysis.

7.5 Safety Measures

Safety and tolerability of treatment will be assessed by vital signs, laboratory assessments, adverse events, and serious adverse events for the safety population. Adverse events will be graded by the NCI CTCAE version 4.03. Categorical measures will be summarized using frequencies and percentages while continuous variables will be summarized using mean, standard deviation, median, minimum, and maximum.

8 STUDY MANAGEMENT

8.1 Data Management

For the purposes of this study at M. D. Anderson Cancer Center, Prometheus will be employed as the data management system. All patients will be registered in CORe before any study specific tests are performed. A brief explanation for required but missing data should be recorded as a comment. The investigator is required to retain, in a confidential manner, the data pertinent to the study for the duration of the study or the maximum period required by applicable regulations and guidelines or institutional procedures. If the investigator withdraws from the study (e.g., relocation, retirement), the records shall be transferred to a mutually agreed upon designee (e.g., another investigator or IRB).

8.2 Posting of Information on Clinicaltrials.gov

Study information from this protocol will be posted on clinicaltrials.gov before enrollment of subjects begins.

8.3 Records Retention

Following closure of the study, the investigator must maintain all site study records (except for those required by local regulations to be maintained elsewhere) in a safe and secure location. The records must be easily accessible when needed and must be available for review in conjunction with assessment of the facility, supporting systems, and relevant site staff.

9 REFERENCES

1. Davies MA, Liu P, McIntyre S, et al. Prognostic factors for survival in melanoma patients with brain metastases. *Cancer* 2011; **117**(8): 1687-96.
2. Topalian SL, Hodi FS, Brahmer JR, et al. Safety, activity, and immune correlates of anti-PD-1 antibody in cancer. *The New England journal of medicine* 2012; **366**(26): 2443-54.
3. Wolchok JD, Yang AS, Weber JS. Immune regulatory antibodies: are they the next advance? *Cancer journal (Sudbury, Mass)* 2010; **16**(4): 311-7.
4. Terme M, Ullrich E, Aymeric L, et al. IL-18 induces PD-1-dependent immunosuppression in cancer. *Cancer research* 2011; **71**(16): 5393-9.
5. !!! INVALID CITATION !!! {}.
6. Pentcheva-Hoang T, Chen L, Pardoll DM, Allison JP. Programmed death-1 concentration at the immunological synapse is determined by ligand affinity and availability. *Proceedings of the National Academy of Sciences of the United States of America* 2007; **104**(45): 17765-70.
7. Hino R, Kabashima K, Kato Y, et al. Tumor cell expression of programmed cell death-1 ligand 1 is a prognostic factor for malignant melanoma. *Cancer* 2010; **116**(7): 1757-66.
8. Azuma T, Yao S, Zhu G, Flies AS, Flies SJ, Chen L. B7-H1 is a ubiquitous antiapoptotic receptor on cancer cells. *Blood* 2008; **111**(7): 3635-43.
9. Dong H, Strome SE, Salomao DR, et al. Tumor-associated B7-H1 promotes T-cell apoptosis: a potential mechanism of immune evasion. *Nature medicine* 2002; **8**(8): 793-800.
10. Hirano F, Kaneko K, Tamura H, et al. Blockade of B7-H1 and PD-1 by monoclonal antibodies potentiates cancer therapeutic immunity. *Cancer research* 2005; **65**(3): 1089-96.
11. Topalian SL, Sznol M, McDermott DF, et al. Survival, durable tumor remission, and long-term safety in patients with advanced melanoma receiving nivolumab. *Journal of clinical oncology : official journal of the American Society of Clinical Oncology* 2014; **32**(10): 1020-30.
12. Zagouri F, Sergentanis TN, Bartsch R, et al. Intrathecal administration of trastuzumab for the treatment of meningeal carcinomatosis in HER2-positive metastatic breast cancer: a systematic review and pooled analysis. *Breast cancer research and treatment* 2013; **139**(1): 13-22.
13. Park WY, Kim HJ, Kim K, et al. Intrathecal Trastuzumab Treatment in Patients with Breast Cancer and Leptomeningeal Carcinomatosis. *Cancer research and treatment : official journal of Korean Cancer Association* 2015.
14. Rubenstein JL, Fridlyand J, Abrey L, et al. Phase I study of intraventricular administration of rituximab in patients with recurrent CNS and intraocular lymphoma. *Journal of clinical oncology : official journal of the American Society of Clinical Oncology* 2007; **25**(11): 1350-6.
15. Perissinotti AJ, Reeves DJ. Role of intrathecal rituximab and trastuzumab in the management of leptomeningeal carcinomatosis. *Ann Pharmacother* 2010; **44**(10): 1633-40.
16. Papadopoulos N, Gerber DL, Eton O, Bedikian AY. The role of intrathecal (IT) use of interleukin-2 (IL-2) in the treatment of leptomeningeal disease (LMD) in patients (pts) with melanoma., Proc. Am Soc Clin Oncol, 21:353a, 2002
17. Peyrl A, Chocholous M, Azizi AA, et al. Safety of Ommaya reservoirs in children with brain tumors: a 20-year experience with 5472 intraventricular drug administrations in 98 patients. *Journal of neuro-oncology* 2014; **120**(1): 139-45.
18. Sandberg DI, Bilsky MH, Souweidane MM, Bzdil J, Gutin PH. Ommaya reservoirs for the treatment of leptomeningeal metastases. *Neurosurgery* 2000; **47**(1): 49-54; discussion -5.
19. Szvalb AD, Raad, II, Weinberg JS, Suki D, Mayer R, Viola GM. Ommaya reservoir-related infections: clinical manifestations and treatment outcomes. *The Journal of infection* 2014; **68**(3): 216-24.

20. Yuan Ji, Ping Liu, Yisheng Li, Nebiyou Bekele B. A modified toxicity probability interval method for dose-finding trials. *Clinical Trials* 2010; **7**(6): 653-63.
21. Chamberlain MC, Glantz M, Groves MD, Wilson WH. Diagnostic tools for neoplastic meningitis: detecting disease, identifying patient risk, and determining benefit of treatment. *Seminars in oncology* 2009; **36**(4 Suppl 2): S35-45.
22. Chamberlain MC. Leptomeningeal metastasis. *Seminars in neurology* 2010; **30**(3): 236-44.
23. Chamberlain M, Soffietti R, Raizer J, et al. Leptomeningeal metastasis: a Response Assessment in Neuro-Oncology critical review of endpoints and response criteria of published randomized clinical trials. *Neuro-oncology* 2014.

10. APPENDICES

10.1. RECIST 1.1 Criteria

Measurability of tumor lesions at baseline

Measurable lesion:

- A non nodal lesion that can be accurately measured in at least one dimension (longest dimension) of
- ≥10 mm with MRI or CT when the scan slice thickness is no greater than 5mm. If the slice thickness is greater than 5mm, the minimum size of a measurable lesion must be at least double the slice thickness (e.g., if the slice thickness is 10 mm, a measurable lesion must be ≥20 mm).
- ≥10 mm caliper/ruler measurement by clinical exam or medical photography.
- ≥20 mm by chest x-ray.

Additionally lymph nodes can be considered pathologically enlarged and measurable if

- ≥15mm in the short axis when assessed by CT or MRI (slice thickness recommended to be no more than 5mm). At baseline and follow-up, only the short axis will be measured [Eisenhauer, 2009].

Non-measurable lesion:

All other lesions including lesions too small to be considered measurable (longest diameter <10 mm or pathological lymph nodes with ≥ 10 mm and <15 mm short axis) as well as truly non-measurable lesions, which include: leptomeningeal disease, ascites, pleural or pericardial effusions, inflammatory breast disease, lymphangitic involvement of the skin or lung, abdominal masses/abdominal organomegaly identified by physical exam that is not measurable by reproducible imaging techniques [Eisenhauer, 2009].

Measurable disease:

The presence of at least one measurable lesion. Palpable lesions that are not measurable by radiologic or photographic evaluations may not be utilized as the only measurable lesion.

Non-Measurable only disease:

The presence of only non-measurable lesions.

Specifications by methods of measurements

The same diagnostic method, including use of contrast when applicable, must be used throughout the study to evaluate a lesion.

All measurements should be taken and recorded in millimeters (mm), using a ruler or calipers.

Ultrasound is not a suitable modality of disease assessment. If new lesions are identified by ultrasound, confirmation by CT or MRI is required.

Fluorodeoxyglucose (FDG)-PET is generally not suitable for ongoing assessments of disease. However FDG-PET can be useful in confirming new sites of disease where a positive FDG-PET scans correlates with the new site of disease present on CT/MRI or when a baseline FDG-PET was previously negative for the site of the new lesion. FDG-PET may also be used in lieu of a standard bone scan providing coverage allows interrogation of all likely sites of bone disease and FDG-PET is performed at all assessments.

If PET/CT is performed then the CT component can only be used for standard response assessments if performed to diagnostic quality, which includes the required anatomical coverage and prescribed use of contrast. The method of assessment should be noted as CT on the CRF.

Clinical Examination:

Clinically detected lesions will only be considered measurable when they are superficial (e.g., skin nodules). In the case of skin lesions, documentation by color photography, including a ruler/calipers to measure the size of the lesion, is required.

Contrast enhanced CT with 5mm contiguous slices is recommended.

Minimum size of a measurable baseline lesion should be twice the slice thickness, with a minimum lesion size of 10 mm when the slice thickness is 5 mm. Whenever possible the same scanner should be used. [Eisenhauer, 2009].

X-ray: Should not be used for target lesion measurements owing to poor lesion definition. Lesions on chest X-ray may be considered measurable if they are clearly defined and surrounded by aerated lung; however chest CT is preferred over chest X-ray [Eisenhauer, 2009].

Evaluation of target lesions:

Definitions for assessment of response for target lesion(s) are as follows:

- Complete Response (CR): Disappearance of all target lesions. Any pathological lymph nodes must be <10mm in the short axis.
- Partial Response (PR): At least a 30% decrease in the sum of the diameters of target lesions, taking as a reference, the baseline sum of the diameters (e.g. percent change from baseline).
- Stable Disease (SD): Neither sufficient shrinkage to qualify for PR nor sufficient increase to qualify for progressive disease.
- Progressive Disease (PD): At least a 20% increase in the sum of the diameters of target lesions, taking as a reference, the smallest sum of diameters recorded since the treatment started (e.g. percent change from nadir, where nadir is defined as the smallest sum of diameters recorded since treatment start). In addition, the sum must have an absolute increase from nadir of 5mm.
- Not Applicable (NA): No target lesions at baseline.
- Not Evaluable (NE): Cannot be classified by one of the five preceding definitions.

Note:

If lymph nodes are documented as target lesions the short axis is added into the sum of the diameters (e.g. sum of diameters is the sum of the longest diameters for non-nodal lesions and the short axis for nodal lesions). When lymph nodes decrease to non-pathological size (short axis <10mm) they should still have a measurement reported in order not to overstate progression.

If at a given assessment time point all target lesions identified at baseline are not assessed, sum of the diameters cannot be calculated for purposes of assessing CR, PR, or SD, or for use as the nadir for future assessments. However, the sum of the diameters of the assessed lesions and the percent change from nadir should be calculated to ensure that progression has not been documented. If an assessment of PD cannot be made, the response assessment should be NE.

All lesions (nodal and non-nodal) should have their measurements recorded even when very small (e.g 2 mm). If lesions are present but too small to measure, 5 mm should be recorded and should contribute to the sum of the diameters, unless it is likely that the lesion has disappeared in which case 0 mm should be reported.

If a lesion disappears and reappears at a subsequent time point it should continue to be measured. The response at the time when the lesion reappears will depend upon the status of the other lesions. For example, if the disease had reached a CR status then PD would be documented at the time of reappearance. However, if the response status was PR or SD, the diameter of the reappearing lesion should be added to the remaining diameters and response determined based on percent change from baseline and percent change from nadir.

Evaluation of non-target lesions

- Definitions for assessment of response for non-target lesions are as follows:
- Complete Response (CR): The disappearance of all non-target lesions. All lymph nodes identified as a site of disease at baseline must be non-pathological (e.g. <10 mm short axis).

- Non-CR/Non-PD: The persistence of 1 or more non-target lesion(s) or lymph nodes identified as a site of disease at baseline ≥ 10 mm short axis.
- Progressive Disease (PD): Unequivocal progression of existing non-target lesions.
- Not Applicable (NA): No non-target lesions at baseline.
- Not Evaluable (NE): Cannot be classified by one of the four preceding definitions.

Note:

In the presence of measurable disease, progression on the basis of solely non-target disease requires substantial worsening such that even in the presence of SD or PR in target disease, the overall tumor burden has increased sufficiently to merit discontinuation of therapy.

In the presence of non-measurable only disease consideration should be given to whether or not the increase in overall disease burden is comparable in magnitude to the increase that would be required to declare PD for measurable disease.

Sites of non-target lesions, which are not assessed at a particular time point based on the assessment schedule, should be excluded from the response determination (e.g. non-target response does not have to be "Not Evaluable").

Confirmation of response

To be assigned a status of PR or CR, a confirmatory disease assessment should be performed no less than 4 weeks (28 days) after the criteria for response are first met.

Overall response criteria

Table 12 presents the overall response at an individual time point for all possible combinations of tumor responses in target and non-target lesions with or without the appearance of new lesions for subjects with measurable disease at baseline.

Table 12: Evaluation of Overall Response for Subjects with Measurable Disease at Baseline

Target Lesions	Non-Target Lesions	New Lesions	Overall Response
CR	CR or NA	No	CR
CR	Non-CR/Non-PD or NE	No	PR
PR	Non-PD or NA or NE	No	PR
SD	Non-PD or NA or NE	No	SD
NE	Non-PD or NA or NE	No	NE
PD	Any	Yes or No	PD
Any	PD	Yes or No	PD
Any	Any	Yes	PD

CR=complete response, PR = partial response, SD=stable disease, PD=progressive disease, NA= Not applicable, and NE=Not Evaluable

Note:

Subjects with a global deterioration of health status requiring discontinuation of treatment without objective evidence of disease progression at that time should be classified as having "symptomatic deterioration." Objective response status is determined by evaluations of disease burden. Every effort should be made to document the objective progression even after discontinuation of treatment.

In some circumstances, it may be difficult to distinguish residual disease from normal tissue. When the evaluation of CR depends on this determination, it is recommended that the residual lesion be investigated (fine needle aspirate/biopsy) to confirm the CR.

10.2. RANO-LMD Response criteria

Assessment		Response		Progressive or refractory disease					Stable disease
				Neurological examination defined progression	CSF defined disease progression	Radiologic defined disease progression	Symptoms^		
Neurological exam		Improved		Worse	Stable	Stable	Stable		Stable
CSF									
Cytology (all cancers)		Negative		Negative	Positive (lack consensus)	Negative	Negative		Negative or positive (solid tumors only)
CNS imaging		Definite Improvement		Stable	Stable	Definite worsening	Stable		Stable or equivocally worsening or improved
Symptom assessment		Improved		Worse or stable	Worse or stable	Worse or stable	Worse		Stable

Legend:

CSF cytology negative	Defined as either true negative or atypical
CSF cytology positive	Defined as true positive or suspicious
Stable	Defined as stable or indeterminate
Symptoms^	Stable; no change (-1 to +1 in symptom inventory
	Worse; -2 to -3 in symptom inventory
	Improved; +2 to +3 in symptom inventory

10.3. Guidance on contraception

Acceptable methods for protocols with a teratogenic drug or when there is insufficient information to determine teratogenicity (choose one of the following 3 options)

OPTION 1: Any TWO of the following methods

- Hormonal methods of contraception
- IUD
- Vasectomy
- Tubal Ligation
- A Barrier method (Female or Male Condom with spermicide, Cervical Cap with spermicide, Diaphragm with spermicide)
- OPTION 2: Male condom (with spermicide) and diaphragm
- OPTION 3: Male condom (with spermicide) and cervical cap

UNACCEPTABLE METHODS OF CONTRACEPTION

- Abstinence (including periodic abstinence)
- No method
- Withdrawal
- Rhythm
- Vaginal Sponge
- Any barrier method without spermicide
- Spermicide
- Progestin only pills
- Concomitant use of female and male condom

If spermicide is not available, use of a male condom without spermicide in conjunction with a hormonal method, IUD, or tubal ligation will be acceptable to fulfill this recommendation. Any barrier method when used alone (without spermicide) or the concomitant use of a female and

male condom, are not considered sufficient methods of contraception, as they carry a failure rate of > 1%.

Women of childbearing potential (WOCBP) receiving nivolumab will be instructed to adhere to contraception for a period of 23 weeks after the last dose of investigational product. Men receiving nivolumab and who are sexually active with WOCBP will be instructed to adhere to contraception for a period of 31 weeks after the last dose of investigational product. These durations have been calculated using the upper limit of the half-life for nivolumab (25 days) and are based on the protocol requirement that WOCBP use contraception for 5 half-lives plus 30 days and men who are sexually active with WOCBP use contraception for 5 half-lives plus 90 days.

10.4. Management Algorithms (See Appendix D)

10.5. MoCA and MDASI-BT (See Appendix E and F)

10.6. KPS Definition

KARNOFSKY PERFORMANCE STATUS SCALE
DEFINITIONS RATING (%) CRITERIA

Able to carry on normal activity and to work; No special care needed.	100	Normal no complaints; no evidence of disease.
	90	Able to carry on normal activity; Minor signs or symptoms of disease.
	80	Normal activity with efforts; some signs or symptoms of disease.
Unable to work; able to live at home and care for most personal needs; varying amount of assistance needed.	70	Cares for self; unable to carry on normal activity or to do active work.
	60	Requires occasional assistance, but is able to care for most of his personal needs.
	50	Requires considerable assistance and frequent medical care.
Unable to care for self; Requires equivalent of institutional or hospital care; diseases may be progressing rapidly.	40	Disabled; requires special care and assistance.
	30	Severely disabled; hospital admission is indicated although death not imminent.
	20	Very sick; hospital admission necessary; Active supportive treatment necessary.
	10	Moribund; fatal processes progressing rapidly.
	0	Dead

Oxford Textbook of Palliative Medicine, Oxford University Press. 1993;109.

FUNCTIONAL ASSESSMENT STAGING (FAST)
(Check highest consecutive level of disability.)

1. No difficulty either subjectively or objectively.
2. Complains of forgetting location of objects. Subjective work difficulties.
3. Decreased job functioning evident to co-workers. Difficulty in traveling to new locations. Decreased organizational capacity. *
4. Decreased ability to perform complex task, (e.g., planning dinner for guests, handling personal finances, such as forgetting to pay bills, difficulty marketing, etc.)
5. Requires assistance in choosing proper clothing to wear for the day, season or occasion, (e.g. patient may wear the same clothing repeatedly, unless supervised. *)
6. A) Improperly putting on clothes without assistance or cueing (e.g., may put street clothes on over night cloths, or put shoes on wrong feet, or have difficulty buttoning clothing) (Occasionally or more frequently over the past weeks. *)
   B) Unable to bathe properly (e.g., difficulty adjusting bath-water temperature) (Occasionally or more frequently over the past weeks. *)
   C) Inability to handle mechanics of toileting (e.g., forget to flush the toilet, does not wipe properly or properly dispose of toilet tissue) (Occasionally or more frequently over the past weeks. *)
   D) Urinary incontinence (Occasionally or more frequently over the past weeks. *)
   E) Fecal incontinence (Occasionally or more frequently over the past weeks. *)
7. A) Ability to speak limited to approximately a half a dozen intelligible different words or fewer, in the course of an average day or in the course of an intensive interview.
   B) Speech ability is limited to the use of a single intelligible word in an average day or in the course of an intensive interview (the person may repeat the word over and over.)
   C) Ambulatory ability is lost (cannot walk without personal assistance.)
   D) Cannot sit up without assistance (e.g., the individual will fall over if there are not lateral rests [arms] on the chair.)
   E) Loss of ability to smile.
   F) Loss of ability to hold up head independently.

*Scored primarily on the basis of information obtained from acknowledgeable informant and/or category.
Reisberg, B. Functional assessment staging (FAST). Psychopharmacology Bulletin, 1988; 24:653-659.

10.7. Recommended Adverse Event Recording Guidelines

Recommended Adverse Event Recording Guidelines					
**Attribution**	**Grade 1**	**Grade 2**	**Grade 3**	**Grade 4**	**Grade 5**
**Unrelated**	Phase I	Phase I	Phase I<br>Phase II	Phase I<br>Phase II<br>Phase III	Phase I<br>Phase II<br>Phase III
**Unlikely**	Phase I	Phase I	Phase I<br>Phase II	Phase I<br>Phase II<br>Phase III	Phase I<br>Phase II<br>Phase III
**Possible**	Phase I<br>Phase II	Phase I<br>Phase II<br>Phase III	Phase I<br>Phase II<br>Phase III	Phase I<br>Phase II<br>Phase III	Phase I<br>Phase II<br>Phase III
**Probable**	Phase I<br>Phase II	Phase I<br>Phase II<br>Phase III	Phase I<br>Phase II<br>Phase III	Phase I<br>Phase II<br>Phase III	Phase I<br>Phase II<br>Phase III
**Definitive**	Phase I<br>Phase II	Phase I<br>Phase II<br>Phase III	Phase I<br>Phase II<br>Phase III	Phase I<br>Phase II<br>Phase III	Phase I<br>Phase II<br>Phase III
